# Magnetic and Photoluminescent Sensors Based on Metal-Organic Frameworks Built up from 2-aminoisonicotinate

**DOI:** 10.1038/s41598-020-65687-6

**Published:** 2020-06-01

**Authors:** Antonio A. García-Valdivia, Sonia Pérez-Yáñez, Jose A. García, Belén Fernández, Javier Cepeda, Antonio Rodríguez-Diéguez

**Affiliations:** 1 Departamento de Química Inorgánica, Facultad de Ciencias, Universidad de Granada, 18071 Granada, Spain; 2 Departamento de Química Inorgánica, Facultad de Farmacia, Universidad del País Vasco/Euskal Herriko Unibertsitatea (UPV/EHU), 01006 Vitoria, Spain; 3 Departamento de Física Aplicada II, Facultad de Ciencia y Tecnología, Universidad del País Vasco/Euskal Herriko Unibertsitatea (UPV/EHU), 48940 Leioa, Spain; 4 Institute of Parasitology and Biomedicine “López-Neyra”, CSIC, Av. Conocimiento s/n, 18600 Granada, Spain; 5 Departamento de Química Aplicada, Facultad de Química, Universidad del País Vasco/Euskal Herriko Unibertsitatea (UPV/EHU), 20018 Donostia-San Sebastian, Spain

**Keywords:** Materials science, Sensors and biosensors, Coordination chemistry, Magnetic materials, Metal-organic frameworks, Chemistry, Materials chemistry, Optical materials

## Abstract

In this work, three isostructural metal-organic frameworks based on first row transition metal ions and 2-aminoisonicotinate (2ain) ligands, namely, {[M(μ-2ain)_2_]·DMF}_n_ [M^II^ = Co (**1**), Ni (**2**), Zn (**3**)], are evaluated for their sensing capacity of various solvents and metal ions by monitoring the modulation of their magnetic and photoluminescence properties. The crystal structure consists of an open diamond-like topological 3D framework that leaves huge voids, which allows crystallizing two-fold interpenetrated architecture that still retains large porosity. Magnetic measurements performed on **1** reveal the occurrence of field-induced spin-glass behaviour characterized by a frequency-independent relaxation. Solvent-exchange experiments lead successfully to the replacement of lattice molecules by DMSO and MeOH, which, on its part, show dominating SIM behaviour with low blocking temperatures but substantially high energy barriers for the reversal of the magnetization. Photoluminescence studied at variable temperature on compound **3** show its capacity to provide bright blue emission under UV excitation, which proceeds through a ligand-centred charge transfer mechanism as confirmed by time-dependent DFT calculations. Turn-off and/or shift of the emission is observed for suspensions of **3** in different solvents and aqueous solutions containing metal ions.

## Introduction

The multifunctionalization of metal-organic frameworks (MOFs) has recently become one of the main research strategies of inorganic and materials chemistry to guide the construction of materials with sensing capacities^1–3^. This is a consequence of the capacity of these materials to allow for multiple physical properties which may coexist or even cooperate in a synergistic way^4,5^. As it is well known, MOFs are a class of potentially porous materials comprised of single metal ions or metal ion clusters linked one another by organic ligands to give an extended crystalline architecture^6–8^. The variety of metal ions and organic ligands opens up an infinite number of possible combinations which allow designing MOFs almost at will in such a way that their structure responds to a particular commitment^9–15^. In particular, the rapid detection of toxic species in environmental and ecological systems is gaining increasing interest because of the large overlap existing between residential and surrounding industrial areas, which already causes many diseases in human being and tends to be expanded in near future^16^. For instance, various salts containing Fe^3+^ and Cu^2+^ ions, usually employed in industry, are eventually found in rivers and streams damaging those ecosystems^17^. The same applies for some common solvents referred to as volatile organic compounds (VOCs) that are air and water pollutants and cause severe environmental problems^18^. In this regard, MOFs are good candidates to drive the detection of all above mentioned molecules in liquid media owing to their specific functions bearing on the surface of the pores, since they are known to show interactions able to provide a reversible load/unload on the material and, hence, a significant change in a property^19,20^.

Focusing on the sensor activity of the MOFs, the transduction mechanism by which the material manifests a change in a property when the target analyte is uploaded is undoubtedly a key point. Most of the systems studied so far are based on luminescence detection because, making use of the changes (increase/decrease on the intensity or shift of the emission signal) in the photoluminescence (PL) of a probe MOF provoked by the presence of the analyte, is very desired for its relative ease of use, technical simplicity and broad adaptability^21^. Moreover, PL in MOFs can have multiple origins which proceed through a complex electronic excitation/emission scenario in which different parts of the hybrid structure are involved: ligand centred (LC) and metal centred (MC) luminescence, charge transfers (CT) processes with different electron pathways, such as ligand-to-ligand (LLCT), ligand-to-metal (LMCT), metal-to-ligand (MLCT), or even guest molecules centred (GC) charge transfers)^22^. To that end, a promising strategy argues for the use of organic ligands with strong absorption (usually aromatic molecules with functionalities containing heteroatoms with lone-pairs) combined with metal ions with closed-shell electronic configuration, which avoid non-radiative quenching^23,24^. Although comparatively less explored than PL sensing, a magnetic response dependent on different guest molecules, that is the change of the magnetic molecular properties of the MOF as a consequence of the analyte loaded in the voids, is an already plausible alternative despite the more complex technical requirements implied^25^. MOFs behaving as single-molecule magnets (SMMs) below a blocking temperature (*T*_*B*_) consist of isolated spin carriers with large magnetic anisotropy which present no (or negligible) magnetic ordering by means of weak intermetallic exchange interactions^26–28^. For transition metals, spin-reversal barrier that promotes slow magnetic relaxation is *U* = │*D*│(S^2^ − 1/4), where *D* and *S* stand for the ground state half-integer spin and axial parameter of the zero-field splitting (*zfs*). That is the reason why cobalt(II) systems, with not only high and non-integer ground state (*S* = 3/2) which reduces the probability of the quantum tunnelling of magnetization (QTM) but also large magnetic anisotropy, have been most widely studied during the last years^29–31^.

In our continuous quest for metal-organic materials showing enhanced PL and magnetic properties, such as those recently reported based on aminonicotinic ligands^32–34^, we are now giving a step forward and combining one of the latter properties with the porosity afforded by the family of isostructural MOFs of {[M(μ-2ain)_2_]·DMF}_n_ (where M^II^ = Co, Ni and Zn and 2ain = 2-aminoisonicotinate) formulae. In particular, given that the porous nature of these materials was already confirmed by gas adsorption capacity and the fact that in those previous reports these MOFs crystallized with different solvents occupying the voids^35^, magnetic behaviour of the cobalt(II) counterpart and PL performance of the zinc(II) counterpart have been deeply analysed, focusing on their modulation by solvent-exchange experiments and/or capture of metal ions.

## Results and Discussion

### Structural description of {[M(μ-2ain)_2_]·DMF}_n_ [M^II^ = Co (1), Ni (2), Zn (3)]

**Figure 1:**
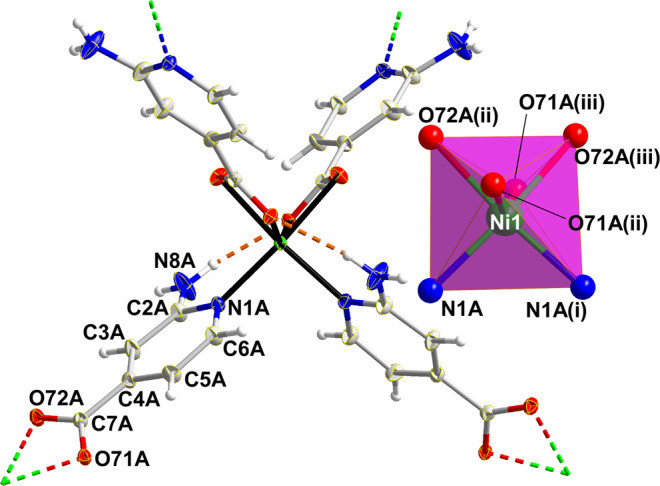
Fragment of crystal structure of compound **2** showing labelling mode and the distorted octahedral coordination environment (inset). Connectivity of the structure is inferred by dashed double-colour lines whereas dashed orange lines stand for hydrogen bonds.

Title compounds are isostructural and crystallize in the orthorhombic *Fddd* space group so their structure will be described using compound **2** as a reference. The crystal structure consists of an entangled 3D open framework. Ni1 exhibits a N_2_O_4_ donor set exerted by its coordination to four symmetry related 2ain ligands by means of two pyridine nitrogen and four carboxylate oxygen atoms (Table 1 and Fig. 1). Given that the latter establish two four-member chelating rings with the metal centre, the resulting coordination polyhedron is severely distorted with regard to a perfect octahedron (S_OC_ = 3.43). It must be highlighted that bond distances are clearly more irregular in the coordination shell of compounds **1** and **3**, which translates into more distorted octahedra (S_OC_ = 4.41 and 4.96 for **1** and **3**, respectively; see ESI).

**Table 1:** Selected bond lengths for all compound.

**Compound 1**					
Co1–N1A	2.073(1)	Co1–O71A(v)	2.314(1)	Co1–O72A(v)	2.060(1)
Co1–N1A(iv)	2.073(1)	Co1–O71A(vi)	2.314(1)	Co1–O72A(vi)	2.060(1)
**Compound 2**					
Ni1–N1A	2.060(1)	Ni1–O71A(ii)	2.075(1)	Ni1–O72A(ii)	2.178(1)
Ni1–N1A(i)	2.060(1)	Ni1–O71A(iii)	2.075(1)	Ni1–O72A(iii)	2.178(1)
**Compound 3**					
Zn1–N1A	2.063(1)	Zn1–O71A(v)	2.034(1)	Zn1–O72A(v)	2.472(1)
Zn1–N1A(iv)	2.063(1)	Zn1–O71A(vi)	2.034(1)	Zn1–O72A(vi)	2.472(1)

[a] Symmetries: (i) –x– 1/4, y,–z + 3/4; (ii) x + 1/4, y + 1/4, –z + 1; (iii) –x – 1/2, y + 7/4, z – 1/4; (iv) –x + 5/4, y,–z + 1/4; (v) x + 7/4, y + 7/4, –z; (vi) –x, y + 7/4, z + 7/4.

**Figure 2:**
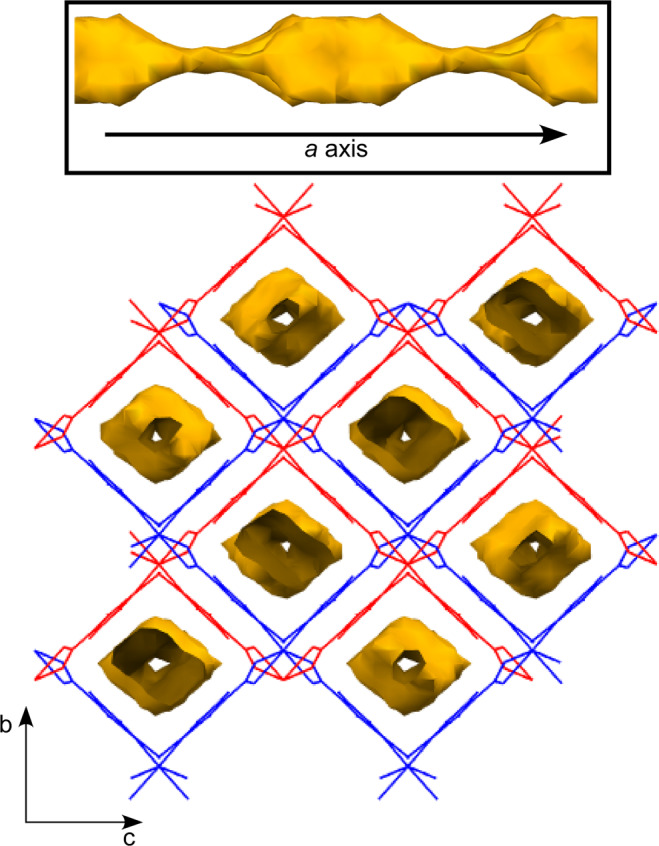
Crystal packing of compound **2** showing the two-fold interpenetrated structure and the microchannels.

2ain anions acquire the μ-*κN1A*:*κ*^*2*^*O71A,O72A* bridging mode in such a way that the central Ni1 atom is connected to four neighbouring ones at a distance of *ca*. 8.77 Å. This coordination mode makes the ligands be somewhat twisted by breaking the planarity of the carboxylate group with respect to the aromatic ring (significantly rotated with an angle of *ca*. 15.9°), which, in turn, gives rise to a tetrahedral building unit from the topological point of view. This arrangement is supported by strong N–H···O hydrogen bonds established among amino and carboxylate groups of 2ain ligands. The junction of building units leads to an open 3D framework of **dia** topological class and (6^6^) point symbol^36,37^ which contains very large cavities where a sphere of 8 Å fits in within (Fig. S2). Nonetheless, the occurrence of such a large free volume allows the crystallization of an identical subnet, which drops the porosity of the eventual doubly-interpenetrated framework to a 36.1% of the unit cell volume (Fig. 2). Both subnets are mutually sustained by means of hydrogen bonding interactions among the exocyclic amino and carboxylate groups belonging to different subnets (see Table S1 and Fig. S3). These supramolecular interactions allows for the occurrence of a stable porous system which consists of narrow microchannels running along the crystallographic *a* axis. Despite the large disorder affecting the lattice solvent molecules occupying the voids, a careful analysis by both TGA/DTA and SQUEEZE routine results confirm that the content of the voids may be determined as one DMF molecule per formula unit (see sections S4 and S5 in the ESI for further detail)^38^. A further analysis of the compound by means of thermodiffractometry shows that the release of solvent molecules does not bring any relevant structural change, as confirmed by the similar shape of the diffractograms recorded according to the increasing temperature.

### Static magnetic properties

Variable temperature dependence of the magnetic susceptibility data were analysed in the 2−300 K range on polycrystalline samples of compounds **1** and **2**. Room temperature *χ*_*M*_*T* product of **1** is 3.22 cm^3^ K mol^–1^, which is significantly higher than that expected for a magnetically isolated spin triplet (*g* = 2.01) in octahedral coordination geometry (1.87 cm^3^ K mol^–1^). Upon cooling, *χ*_*M*_*T* value experiments a slight and progressive decrease up to 50 K, below which it subtly drops off to reach 1.85 cm^3^ K mol^–1^ at low temperature. This behaviour may be mainly attributed to the *zfs* that may cause a high intrinsic magnetic anisotropy arising from the first order spin-orbit coupling (SOC) usually present in Co(II) atoms derived from its ^4^T_1g_ ground state in high-spin octahedral geometry^39,40^, though the occurrence of weak antiferromagnetic interactions cannot be discarded. Taking into account the absence of an appropriate mathematical expression to estimate the nature of the magnetic interactions for 3D networks containing cobalt(II) ions, the data were analysed with the Curie-Weiss law. Given the fact that compound **1** follows Curie−Weiss law in the whole temperature range, *χ*_*M*_^−1^ vs T was fitted giving the results shown in Table 2 (see also ESI). Moreover, the data were also fitted to the phenomenological equation proposed by Rueff and co-workers^41^ (Eq. 1) in view of the SOC present, from which the antiferromagnetic exchange interactions were estimated:

1 $${\chi }_{M}T=A\,\exp (\text{-}{E}_{1}/\kappa T)+B\,\exp (\text{-}{E}_{2}/\kappa T)$$

**Table 2:** Best fitting results for compound **1**.

Curie-Weiss law fitting^a^							
*C*	3.27	*θ*	–7.52 K				
Rueff phenomenological fitting (Eq. 1)							
*A*	0.93(4)	*B*	2.34(5)	*E*_*1*_*/κ*	23(2)	–*E*_*2*_*/κ*	–0.80(9)
Hamiltonian SOC (Eq. 2)^b^							
*λ*	–110	*σ*	–1.12	*Δ*	188	*g*	2.10
Hamiltonian *zfs* (Eq. 3)^c^							
*g*_*x*_ */ g*_*y*_	2.36	*g*_*z*_	3.26	*D*	–11.9		

[a] Units: *C* constant and *θ* are given in cm^3^ K mol^−1^ and K, respectively. [b] *λ* and *Δ* parameters are expressed in cm^–1^. [c] *D* parameter is given in cm^–1^.

The fact that the sum of *A* and *B* parameters equals the Curie constant and that “activation energies” of SOC (*E*_*1*_) and (*E*_*2*_) exchange interactions (see Table 2) fall in the range of related Co(II) compounds^42^, weak antiferromagnetic interactions may be claimed to occur among Co(II) ions in the 3D network.

**Figure 3:**
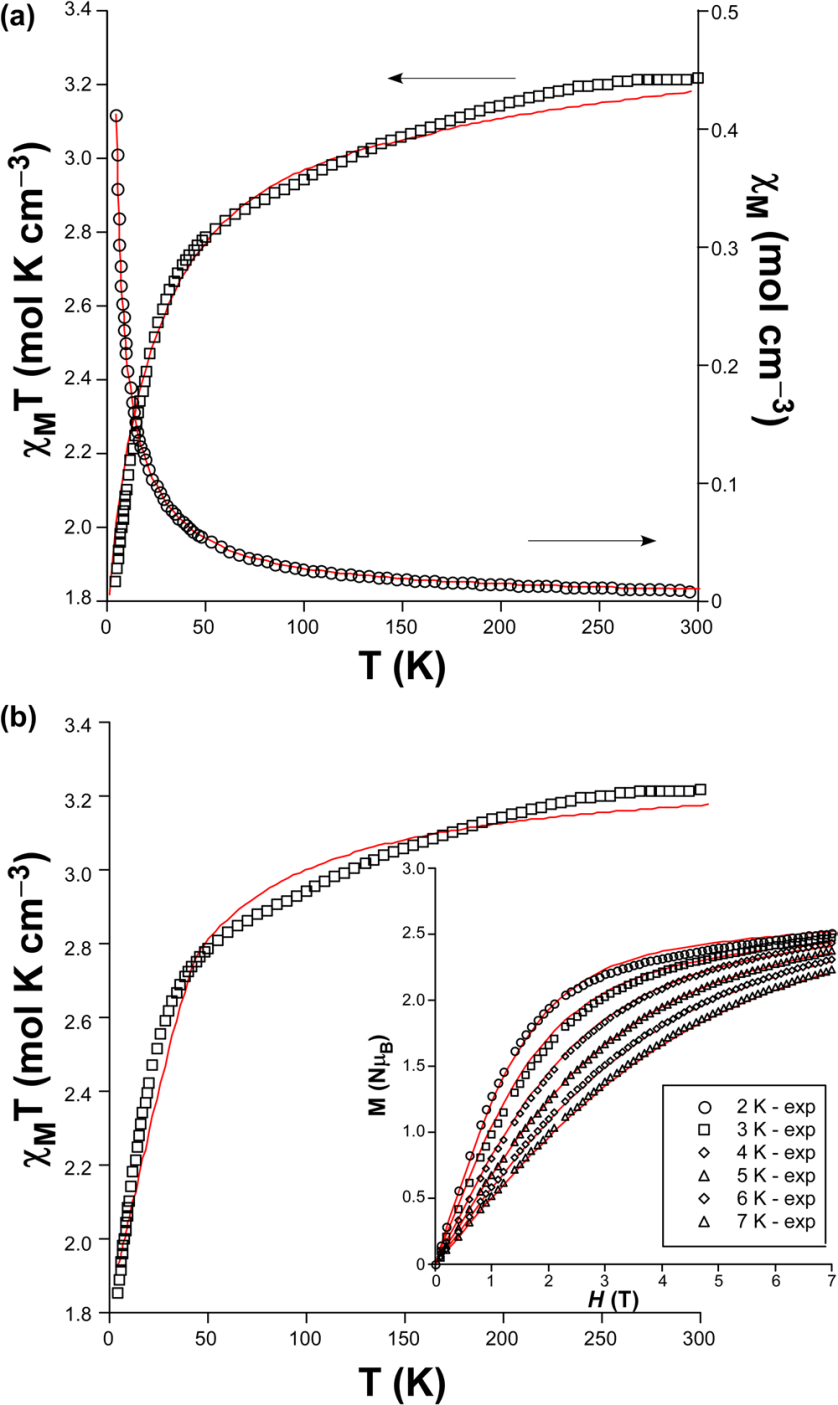
(**a**) *χ*_*M*_ (o) *and χ*_*M*_*T* (◻) vs *T* plots of **1** with best fit according to Eq. 2. **(b)** Simultaneous fitting of the *χ*_*M*_*T* vs *T* and *M* vs *H* (inset) plots using Eq. 3.

DFT calculations performed on a suitable model of compound **1** (see Fig. S21) give a value of the coupling constant (*J*) of –0.07 cm^–1^, which concords well with the mentioned negligible magnetic interactions. Accordingly, cobalt(II) spin carriers may be considered to be isolated by the regular bridging ligands, bearing in mind that the shortest distance among them is of about 8.8 Å, thus allowing us to analyse the SOC effects by means of fitting of the magnetic susceptibility with the Hamiltonian given in Eq. 2^43^:

2 $$\hat{H}=\sigma \lambda ({L}_{Co}{S}_{Co})+\varDelta [{{L}_{z,Co}}^{2}\mbox{--}{L}_{Co}({L}_{Co}+1)/3+{\mu }_{B}H\cdot (\mbox{--}\sigma {L}_{Co}+g{S}_{Co})$$

where all parameter have their usual meaning. The calculated curve using the PHI program^44^ reproduces quite well the experimental one, though a slight deviation is found mainly for the high temperature data (see Table 2 and Fig. 3a).

Among the estimated parameters, it deserves to be mentioned the large and positive value of *Δ*, which suggests that only the two lowest Kramers doublets of the ^4^A_2_ ground term are thermally populated, in turn meaning that the axial *zfs* within the quartet state matches well with the energy gap existing between them. Therefore, the magnetic properties may be interpreted by means of the spin Hamiltonian of Eq. 3 (Fig. 3b):

3 $$\hat{H}=D({{\hat{S}}_{z}}^{2}-\hat{S}/3)+E({{\hat{S}}_{x}}^{2}+{{\hat{S}}_{y}}^{2})+{\mu }_{B}\overline{B}\cdot g\cdot \hat{S}$$

in which *S* is the spin of the ground state (*S* = 3/2), *D* and *E* are the axial and rhombic magnetic anisotropies, and *H* is the applied magnetic field. On its part, magnetization curves collected at several temperatures (2–7 K) under an applied field ranging from 0 to 7 T do not reach the theoretical saturation for S = 3/2 (M_sat_ = 3.3, with g = 2.2), but they show a value of ca. 2.45 Nμ_B_ at 2 K. This behaviour together with the fact that isothermal curves do not collapse in a single master curve are indicative of magnetic anisotropy. A simultaneous fitting of susceptibility and magnetization data with PHI using Eq. 3 gives *D* = –16.1 cm^–1^, *E* = –0.1 cm^–1^, and g = 2.29, whereas the fitting was substantially improved by allowing a slightly anisotropic gyromagnetic tensor, such that the values found in Table 2 were achieved with an R = 8.5 × 10^–4^. Assuming an axial anisotropy, being *E* ≈ 0, the energy separation between ± 1/2 and ± 3/2 doublets equals 2*D* due to the second-order SOC present in the distorted octahedral Co(II) ion.

**Figure 4:**
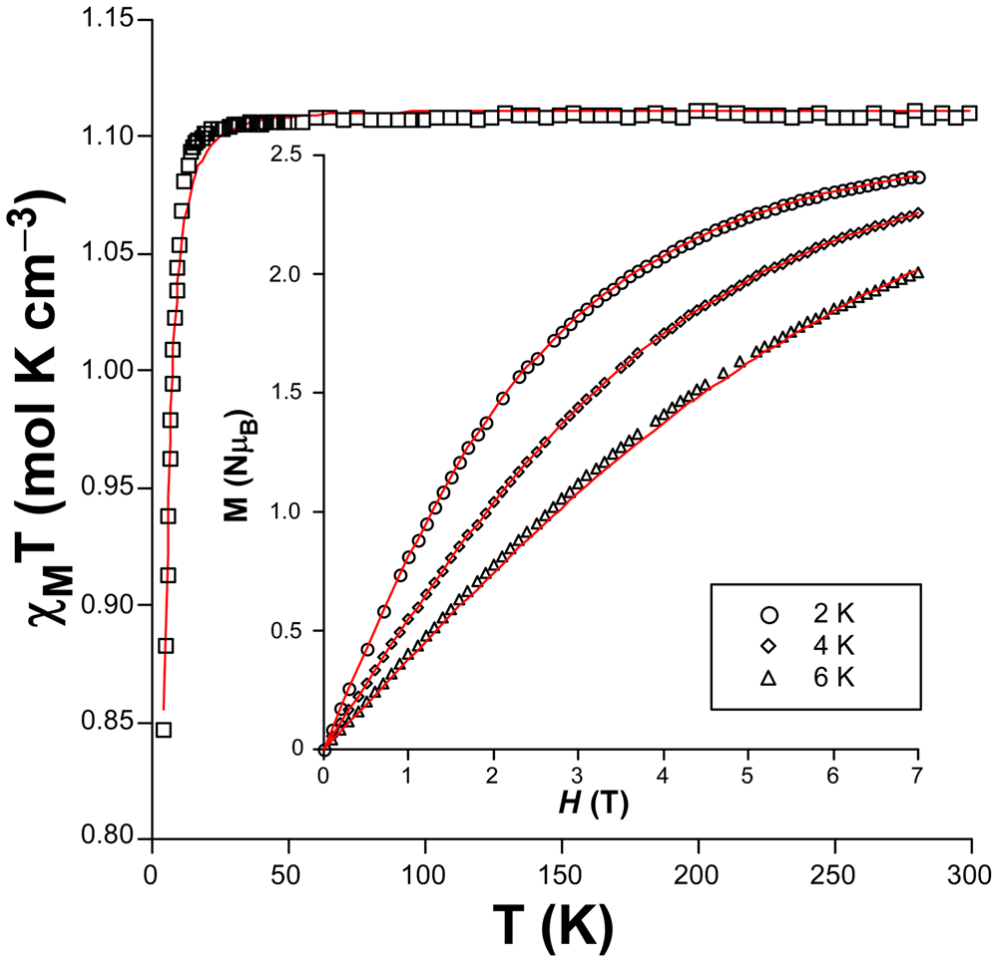
Simultaneous fitting of the *χ*_*M*_*T* vs *T* and *M* vs *H* (inset) plots using Eq. 3 for compound **2**.

*χ*_*M*_*T* vs *T* plot of compound **2** shows a room temperature *χ*_*M*_*T* value (1.11 cm^3^ mol^−1^ K) close to that expected for an isolated ion (1.00 cm^3^ mol^−1^ K with g = 2.01, see Fig. 4). This curve shows a plateau from room temperature up to 25 K, where it experiments an abrupt drop up to 0.85 cm^3^ K mol^–1^ at 5 K, probably due to the occurrence of *zfs* and weak antiferromagnetic interactions, which may be assumed given that the shortest 2ain mediated Ni···Ni distance along the network is of the same order of the cobalt(II) counterpart (larger than 8.7 Å) and the absence of significant π-π stacking interactions between the subnets (where Ni···Ni separations of ca. 8 Å are found). In this sense, the computed broken symmetry procedure upon model 2 supports the weak antiferromagnetic nature of the intramolecular exchange interaction (*J* = –0.55). Accordingly, the experimental *χ*_*M*_*T* vs *T* data were fitted with the Hamiltonian shown in Eq. 3 with PHI, from which the following set of parameters were achieved: *g*_iso_ = 2.11 and *D* = –4.4 cm^–1^ (with a negligible value of *E* < 0.1 cm^–1^) with R = 4.6 × 10^–5^. It is worth noticing that the value of *D* lies within the range found for similar octahedral Ni^II^ complexes^45^.

### Dynamic magnetic properties

**Figure 5:**
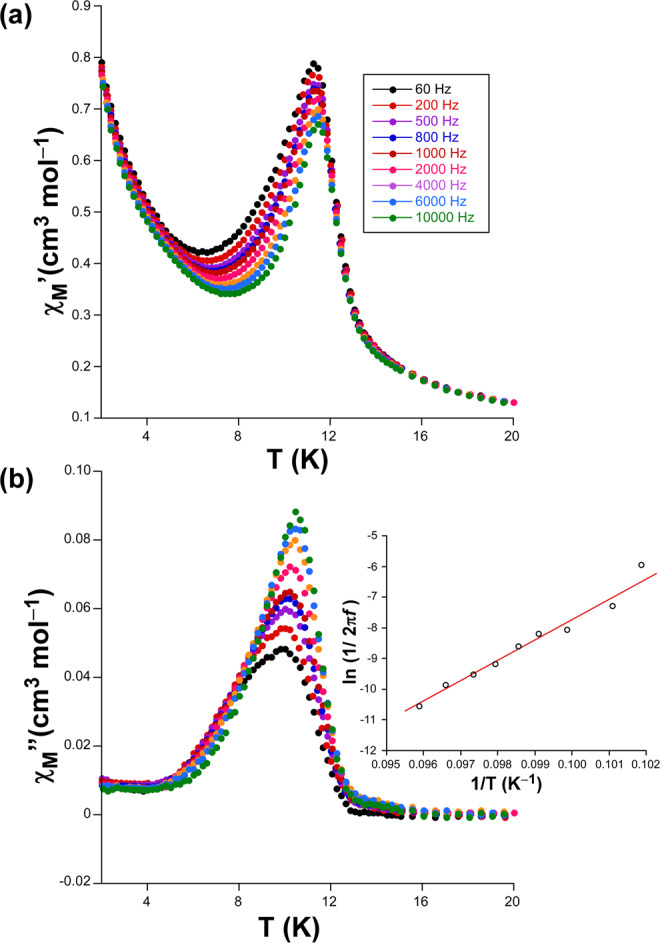
Temperature dependence of the **(a)** *χ*_*M*_′ and **(b)** *χ*_*M*_″ signals for compound **1** under an applied field of 1000 Oe. Inset shows the Arrhenius plot with the linear fitting to estimate the thermal barrier for the reversal of the magnetization.

The magnetic anisotropy found for compounds **1** and **2** prompted us to study their spin dynamics by means of *ac* magnetic susceptibility measurements (using an alternating field of 3.5 Oe). Compound **1** exhibited a slight frequency-dependent signal although the maxima remained below 2 K when applying a zero *dc* field, which did not allow further fitting of the data (Fig. S16). This behaviour seems to indicate that magnetic relaxation proceeds through a fast quantum tunnelling (QTM) derived from intramolecular and/or strong hyperfine interactions occurring with the *I* = 7/2 nuclear spin of the Co(II) atom^29d,^^46^. Interestingly, when a *dc* field of 1 kOe is applied, compound 1 shows temperature-dependent in-phase (*χ*_*M*_′) and out-of-phase (*χ*_M_″) signals (Fig. 5), whereas QTM could not be suppressed for compound **2** so no frequency dependence was observed. A first inspection of the *ac* data of **1** reveals that *χ*_*M*_″ signals peaking at ca. 10 K present a remarkable width, mainly for the low frequency regime (60–1000 Hz), which makes one suspect about the occurrence of two consecutive and overlapped maxima. In any case, the most remarkable feature of the *ac* signals is clearly the fact both peaks (*χ*_*M*_*’* and *χ*_*M*_″) are weakly dependent of the frequency. In fact, the frequency shift calculated as *ϕ* = Δ*T*_*p*_/[*T*_*p*_Δ(log *f*)] (where *T*_*p*_ corresponds to the peak of *χ*_*M*_″(*T*) curve and *f* to the frequency) gives a low value of 0.03, which is a common value for spin glasses (*ϕ* <0.1)^47,48^. Accordingly, relaxation times (*τ*) estimated from the *τ* = 1/(2 π *f*_max_) expression based on *χ*_*M*_″(*T*) peak give thermally activated relaxations with τ_0_ = 9.4 ×10^–33^ s and *U* = 660 K, values that agree well with those recorded for other reported spin-glass materials with slow dynamics^49,50^. At this point, it must be highlighted that this sort of glass-like magnetic behaviour is usually related to a field-dependence behaviour (derived from canted antiferromagnetism or long-ranged magnetic ordering) which, yet not observed in *dc* measurements, could be the present case in view of the chiral structure being comprised of two 3D sublattices. However, the fact that a weak SIM behaviour could be overlapped by the dominating glassy-state, in view of the width of the signal, is not to be fully discarded.

### Solvent-dependent magnetic behaviour of compound 1

**Figure 6:**
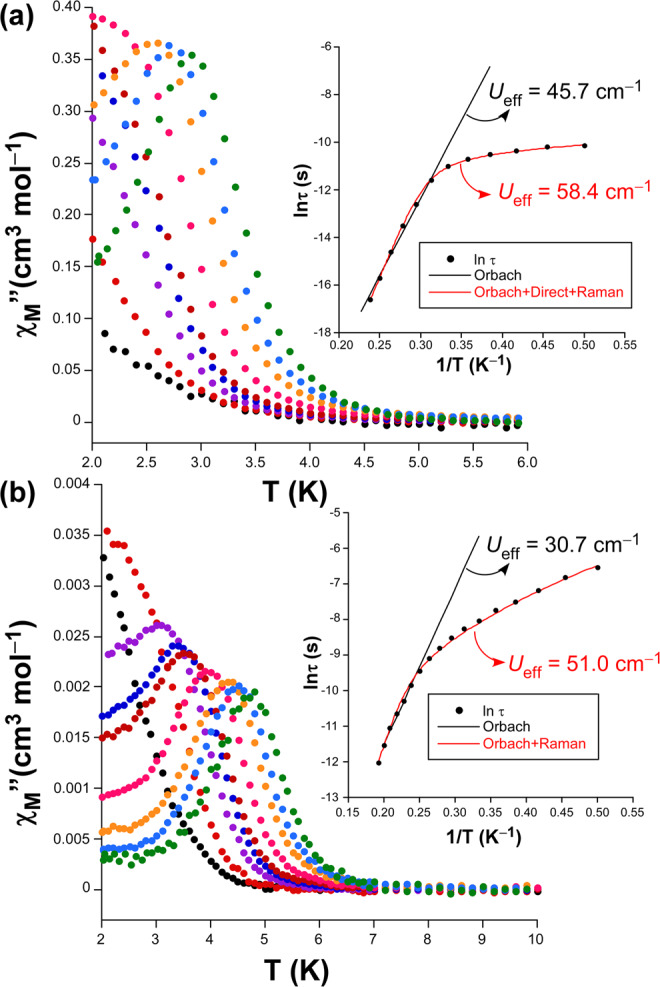
Temperature dependence of the *χ*_*M*_″ signals and best fitting results for the relaxation times for compounds (**a**) **1-DMSO** and (**b**) **1-MeOH**.

Solvent-exchange experiments were accomplished upon polycrystalline sample of compound **1** in view of its intriguing magnetic behaviour and potentially porous nature, which could a priori endow the material with a guest (solvent)-dependent magnetism. Two different solvents, such as DMSO and MeOH, were selected not only for their common use in the synthesis of MOFs but also for their hazardous nature. The exchange of lattice solvent molecules was successfully achieved by immersing fresh sample of **1** into 10 mL of the solvent and letting it to stand for two days under a soft stirring, which led to the isomorphous compounds {[Co(μ-2ain)_2_]·2MeOH}_n_ (**1-MeOH**) and {[Co(μ-2ain)_2_]·1.5DMSO}_n_ (**1-DMSO**). Note that the proposed formula was confirmed by elemental analyses, ICP/AES, and TG/DTA experiments (see ESI). Though both compounds retain the crystalline framework to a large extent, they experience some slight changes due to the replacement of the pore molecules. A careful evaluation of the cell parameters shows a common trend: *a* and *b* axes are shrunk whereas *c* is stretched. This behaviour seems to indicate that pore channels are somewhat crushed when replacing the DMF molecules by DMSO and MeOH, respectively for **1-DMSO** and **1-MeOH**, a fact that points to a relative displacement of the subnetworks. The analysis of the *dc* properties of the exchanged MOFs reveals a similar magnetic behaviour with progressive decrease of the *χ*_*M*_*T* product as the temperature drops. Nonetheless, a larger magnetic anisotropy may be inferred from the steepest decrease of *χ*_*M*_*T* in **1-DMSO** compared to **1-MeOH** together with the larger separation between magnetization curves. In fact, mathematical fitting of the data with above mentioned Eqs. 2 and 3 come to the same conclusion supporting a small increase of the axial parameter (*D* = –20 for **1-DMSO** and –16 cm^–1^ for **1-MeOH**, compared to –11.9 cm^–1^ for **1**). Even more exciting is the fact that such an increase in the anisotropy is accompanied by a deep change of the magnetic nature of the materials, since they can be now referred to as SIMs^51^ under an external *dc* of 1000 Oe (no signal is observed with zero field) given their strong frequency-dependent *χ*_*M*_″ signal. Moreover, it must be also noticed that these maxima become narrower than those shown by the neat compound (Fig. 6).

At first sight, the blocking temperature (below which the compounds behave as a SIM) drops down in both cases, among which **1-DMSO** presents well-defined maxima only those curves with an oscillating frequency above 1000 Hz. Instead, the maxima are much closer to each other for **1-DMSO** rather than **1-MeOH**, from which it is deduced that a faster magnetic relaxation occurs in the former. The Cole-Cole plots below 3.4 K for **1-DMSO** and 4.2 K for **1-MeOH** can be well fitted by a generalized Debye function, which it is an important difference with respect to double semicircles shown by pristine compound **1**, where α values ranging in 0.17–0.29 and 0.10–0.26 are found, respectively for **1-DMSO** and **1-MeOH** (Figs. S18 and 19). These values, implying a wide distribution of the relaxation times, are indicative of the occurrence of various mechanisms in the relaxation of the magnetization. In fact, Arrhenius plots in the form of ln(*τ*) vs *T*^–1^ deviate from linearity at low temperature in both cases. Fitting of the high temperature data by means of Orbach process gives values of the effective barrier and pre-exponential factors of *U*_*eff*_ = 65.3 K (45.7 cm^–1^) and τ_0_ = 1.36 ×10^–12^ s for **1-DMSO** and 43.9 K (30.7 cm^–1^) and τ_0_ = 1.54 ×10^–9^ s for **1-MeOH**, which are somewhat higher than those reported for most of polymeric metal-organic compounds behaving as SIMs^26^. Note also that these energy barriers agree with the expected energy separation between Kramers doublet (2*D* = 32 ≈ 30.7 cm^–1^ for **1-MeOH** and 2*D* = 40 ≈ 45.7 cm^–1^ for **1-DMSO**). However, it must be highlighted that the rise of the energy barrier is linked to the relaxation rate, among which the τ_0_ of 1.36 × 10^–12^ s estimated for **1-DMSO** clearly exceeds the usual range (between 10^–6^ – 1 ×10^–11^ s) attributed to Co(II)-based SIMs. This fact that comes to conclude that the exchange of DMSO in the pores modifies the disposition of the subnetworks such that spin carriers can probably interact through intermolecular interactions, explaining the more abrupt drop in the *χ*_*M*_*T* vs *T* curve (see Fig. S15). On another level, very reliable fittings were achieved by combining the Orbach with Raman and/or direct relaxation processes (Eq. 4 and/or 5), which have previously been employed successfully in the analysis of Co(II)-based compounds with related coordination environment^32d,^^52^.

4 $${\tau }^{\mbox{--}1}={A}_{direct}T+{B}_{Raman}{T}^{n}+{{\tau }_{0}}^{\mbox{--}1}\exp (\mbox{--}{U}_{eff}/{\kappa }_{B}T)$$

5 $${\tau }^{\mbox{--}1}={B}_{Raman}{T}^{n}+{{\tau }_{0}}^{\mbox{--}1}\exp (\mbox{--}{U}_{eff}/{\kappa }_{B}T)$$

Best fitting with the multiple relaxation processes gives *U*_*eff*_ = 83.4 K (58.4 cm^–1^), τ_0_ = 1.45 × 10^–14^ s, A_direct_ = 1190(30) s^–1^ K^–1^, *B*_*Raman*_ = 4.1(2) s^–1^, *n* = 7.8(1) for **1-DMSO** and *U*_*eff*_ = 72.8 K (51.0 cm^–1^), τ_0_ = 6.12 ×10^–10^ s, *B*_*Raman*_ = 8.7(2) s^–1^, *n* = 4.1(2) for **1-MeOH**.

### Photoluminescence properties of compound 3

**Figure 7:**
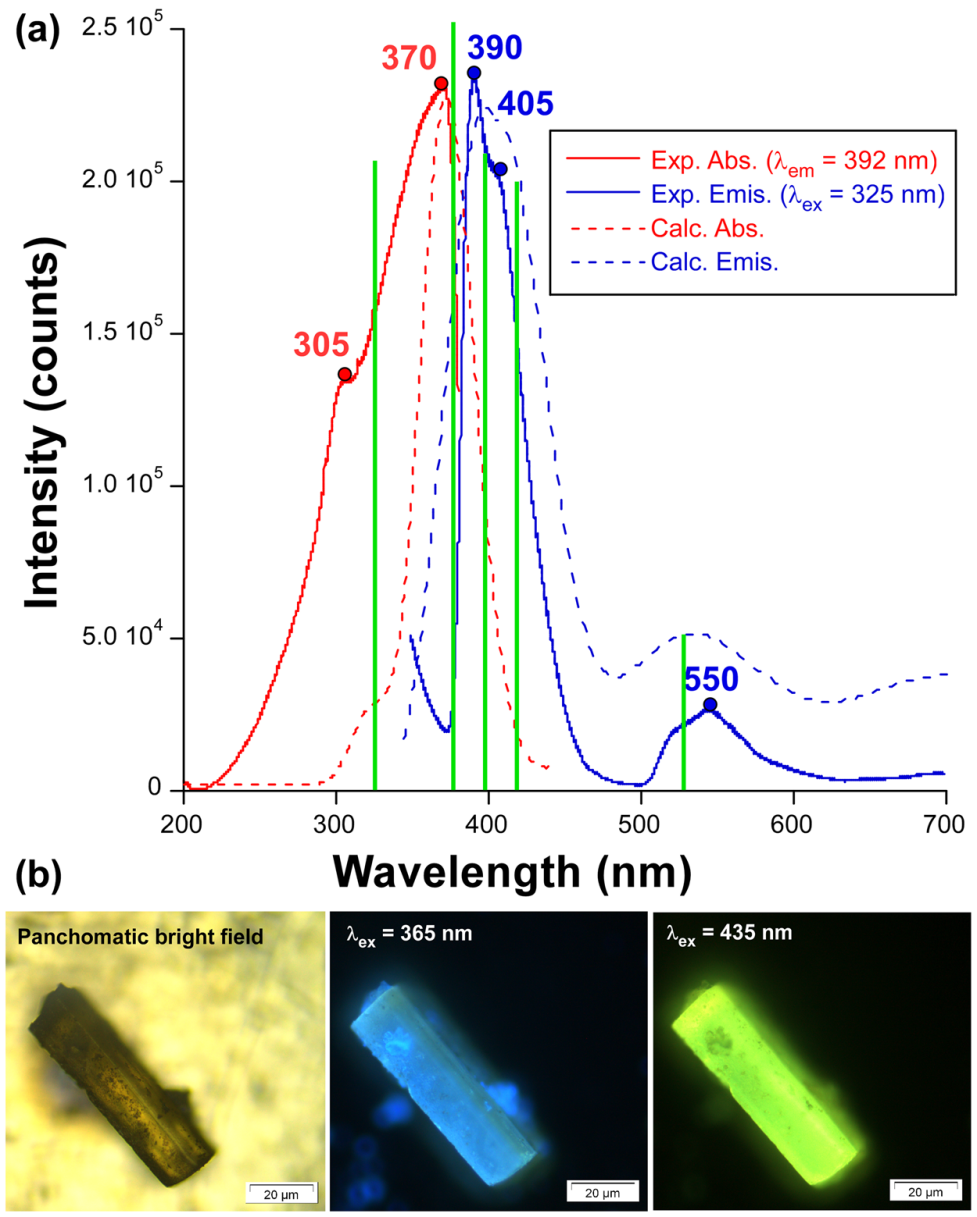
(**a**) Room temperature excitation (red) and emission (blue) spectra of compound **3** showing the most relevant experimental maxima (circles) and calculated (TD-DFT) main vertical excitations (green lines). **(b)** Micro-PL photographs of a single crystal of **3** illuminated with different lights.

Excitation and emission spectra were measured on polycrystalline sample of compound **3** since, being comprised of carboxylic pyridine ligands such as 2ain and metal ions with closed-shell electronic configuration, i.e. Zn(II), it is capable of unveiling interesting photoluminescence in solid state. The emission spectrum under UV light (at the shoulder λ_ex_ = 305 nm) at room temperature (295 K) shows a somewhat narrow band, composed of the maxima (λ_em_ = 390 nm) and a shoulder (λ_em_ = 405 nm), in addition to a wide and weaker band peaking around 550 nm (Fig. 7a). The excitation spectra recorded at the main emission wavelength contains, in addition to the previous shoulder at 305 nm, another main contribution peaking at λ_ex_ = 370 nm, which shows the same PL mechanism in view of the identical emission spectrum achieved at the latter excitation wavelength (Fig. S22). To further investigate the origin of the less energetic band at λ_em_ = 550 nm, an excitation spectrum has been also measured, confirming that it arises from the same excitation path (see Fig. S23). These two bands provide this material, as observed in the images taken on the microscope, with a variable emission consisting of a brilliant blue greenish light under excitation with UV radiation (λ_ex_ = 365 nm) or a lime green emission with a less energetic radiation (λ_ex_ = 435 nm), in such a way that those wavelengths corresponding to the maximum of the emission band are avoided (Fig. 7b). The emission quantum yield measured in absolute terms with an integrated sphere is low (1.75%). The calculated PL spectra conducted on a suitable model of **3** (see Experimental Section) reproduce very well both excitation and emission processes, finding only substantial differences for the relative intensities of the minor bands.

**Figure 8:**
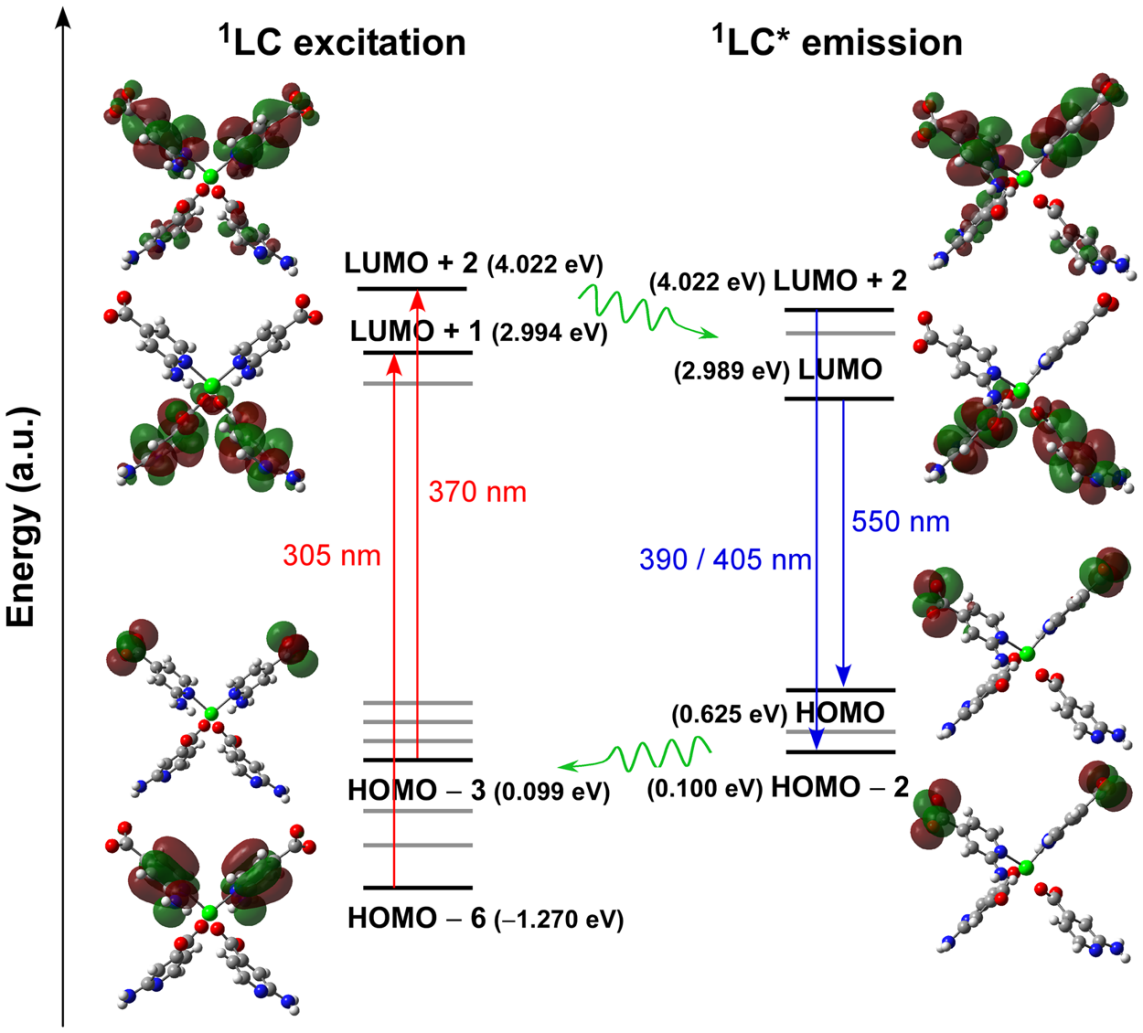
Schematic representation of the most intense excitation (red) and emission (blue) lines of compound **3** with their corresponding MOs. Values given between brackets represent the energies.

Starting from the molecular excitation of the compound, absorption of light at the two main contributions seems to proceed through slightly different electronic transitions (Table 3, note that these are the most intense transitions gathered as a representative sample of the band). On the one hand, the shoulder at 305 nm corresponds to a π → π* transition in which the involved MOs are centred on the aromatic ring of 2ain ligands (Fig. 8), while the band maximum at 370 nm may be better described as a n→π* transition given that HOMO – 2/3 lie over the carboxylate group on the other. On its part, the PL emission takes also place through LCCT mechanism since electrons drop from excited LUMO – n orbitals, of π* nature with lobes extended over the whole molecule or solely the aromatic ring (see Fig. 8), to HOMOs consisting of n or π orbitals based on the carboxylate group for the main (λ_em_ = 390 and 405 nm) or the minor (λ_em_ = 550 nm) bands, respectively.

**Table 3:** Calculated main excitation and emission energies (nm), singlet electronic transitions and associated oscillator strengths of model 3.

Exp. λ	Calcd. λ	Electronic transitons	Osc. strength (a.u.)
**Excitation energies**			
305	318	HOMO – 6 → LUMO + 1(51%) HOMO – 7 → LUMO (49%)	0.083
370	372	HOMO – 3 → LUMO + 2 (51%) HOMO – 2 → LUMO + 3 (42%)	0.112
**Emission energies**			
390	388	HOMO – 2 ← LUMO + 4 (52%) HOMO – 2 ← LUMO + 2 (40%)	0.084
405	408	HOMO – 2 ← LUMO + 2 (89%)	0.081
550	539	HOMO ← LUMO (97%)	0.038

For comparative purposes, emission spectra of **3** were recorded at different temperatures under the same experimental conditions in order to check how the suppression of the vibrational quenching, i.e. the molecular vibrations/motions occurring in the ligand that may be overlapped with the radiative emission and hence draw emission capacity to the system^53^, as the temperature is dropped affects the PL response of the material. Upon cooling the system from RT down to 10 K, the band maximum does not show any remarkable shift although it progressively gains intensity, mainly in the temperature range of 200–150 K where the intensity shows a quantitative leap. To summarize, the emission intensity at λ_max_ ≈ 400 nm is much greater for 10 K data compared to RT (about 55 times larger in terms of emitted integrated intensity, Fig. S25). With the aim of enlarging the temperature-dependent characterization, emission decay curves were also measured at three representative temperatures (295, 150 and 10 K), revealing very similar lifetimes in the range of hundreds of microseconds. In any case, it is worth highlighting that the observed lifetime remains constant along the whole emission spectrum and shows the expected trend with the temperature although the change is somewhat slight (τ being 239(2), 169(4) and 121(3) μs for the above mentioned temperatures, see Table S3), which indicates that a unique PL mechanism is preserved irrespective of the temperature of the system. These short lifetimes contrast when compared to those measured for other systems consisting of Zn(II) and positional isomers of the 2ain ligand, as it is the case of [Zn(µ–6ani)_2_]_n_ and [Zn(µ–2ani)_2_]_n_^32^, which displayed long-lasting phosphorescence (LLP) emissions that could be traced by human eye. As concluded from the analysis of molecular based phosphorescent reported so far^54^, a major reason for the occurrence of LLP in metal-organic compounds with closed-shell ions is attributed to the intermolecular forces established by ligands in the framework, in such a way that strong interactions are able to freeze the molecules and make lowest-lying triplet states (T_1_) more accessible and shielded against quenching. As a matter of fact, the molecular N–H vibrations of the amino group, known to be a main oscillator which enables the non-radiative quenching^55^, seem to be less suppressed in compound **3** compared to their disposition in the above mentioned CPs. In these latter compounds, isomeric 6ani and 2ani ligands establish more rigid hydrogen bonds, particularly in the case of the intramolecular hydrogen bonds found in [Zn(µ−2ani)_2_]_n_, whereas the 2-fold interpenetration brings higher flexibility to the crystal building of **3**, explaining the short lifetimes.

### PL sensing properties

**Figure 9:**
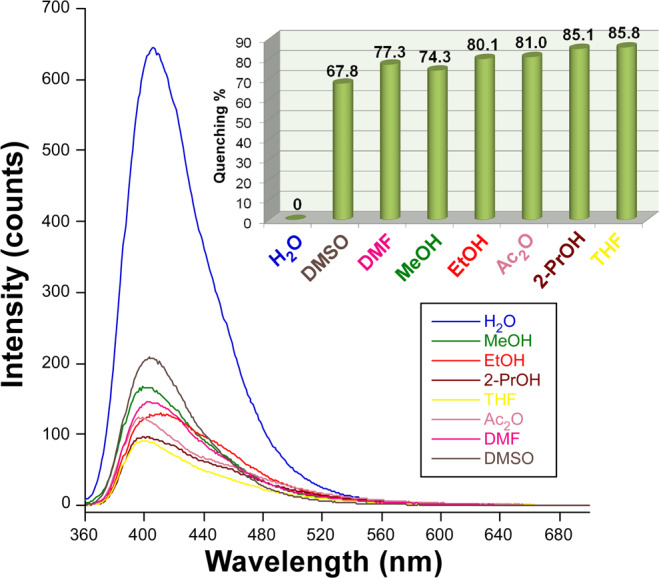
PL emission spectra of dispersion of compound **3** in different solvents at their maximum excitation wavelength. Inset shows quenching percentage estimated from the emission intensity relative to H_2_O@**3** is reflected in the upper bar chart.

The flexible porous nature revealed by compound **3**, consisting of a stable doubly interpenetrated metal-organic framework with pores in which small molecules could be exchanged, in addition to the strong blue greenish PL displayed at RT instigated us to study its performance as PL sensing material for various solvents and metal ions. When polycrystalline sample is dispersed in different solvents, solvent@**3** hereafter, the main emission band experiments not only significant changes regarding the intensity but also slight shifts for the maximum of the emission band (λ_max_). In a first approach, the analysis of the spectra measured at a representative wavelength (λ_ex_ = 350 nm, which falls within the excitation maximum for all suspensions) under the same equipment configuration (identical slit aperture and photomultiplier voltage) revealed that the emission decreases following the expected sequence according to the solvent polarity (note that dielectric constants are shown between brackets): H_2_O (80.1) > DMSO (46.7) > DMF (36.7) > MeOH (32.7) > EtOH (24.5) > Ac_2_O (20.7) > 2-PrOH (19.2) > THF (7.58)^16b,^^56^. In other words, the higher the polarity of the solvent the largest its quenching capacity (see Fig. S28). Moreover, the presence of a minor emission peak, in the form of a shoulder peaking at λ_em_ ≈ 450 nm, was also observed for less polar solvents (Ac_2_O, 2-PrOH and THF), which is probably due to the enabling of an electronic transition arising from a less energetic LUMO level derived from solvation effects^57^. In this sense, it must be recalled that 2ain ligands in the crystal structure possess uncoordinated amino groups exposed to the microchannels, where hydrogen bonding and Van der Waals interactions established with solvent molecules may affect the LCCT mechanism and thus the PL emission^58^. With the aim of getting insights into the potential applicability of this material, a further analysis showed that the main emission follows a solvent-dependent excitation, given the drastic changes shown in the excitation spectra monitored at the λ_max_ (where only one wide band or two narrower maxima are observed depending on the selected solvent, see ESI). A new set of measurements with variable excitation wavelength in order to maximize the solvent-dependent PL behaviour (focusing at the most intense λ_ex_) indicates that **3** keeps a strong PL emission in water (note that water itself brings some quenching compared to solid state but it is employed as a reference for the rest of solvents) whereas, taking water as a reference, the rest of studied solvents cause a large quenching (with a quenching percentage (QP) above 65%, Fig. 9). Taking into account that crystal structure of **3** presents no significant change (as corroborated by PXRD collected for samples recovered from solvent@**3** suspensions, Fig. S31), it may be assumed that solvents exert: (i) a dynamic quenching involving solvation processes on external surface of the particles in the suspension, and (ii) a quenching derived from the particular interactions (enabling competitive absorption and energy transfers)^59^ occurring between solvent molecules and the internal surface of the microchannels, where factors such as size and hydrogen bonding capacity are key and govern the diffusion of solvent molecules throughout the pore system.

**Figure 10:**
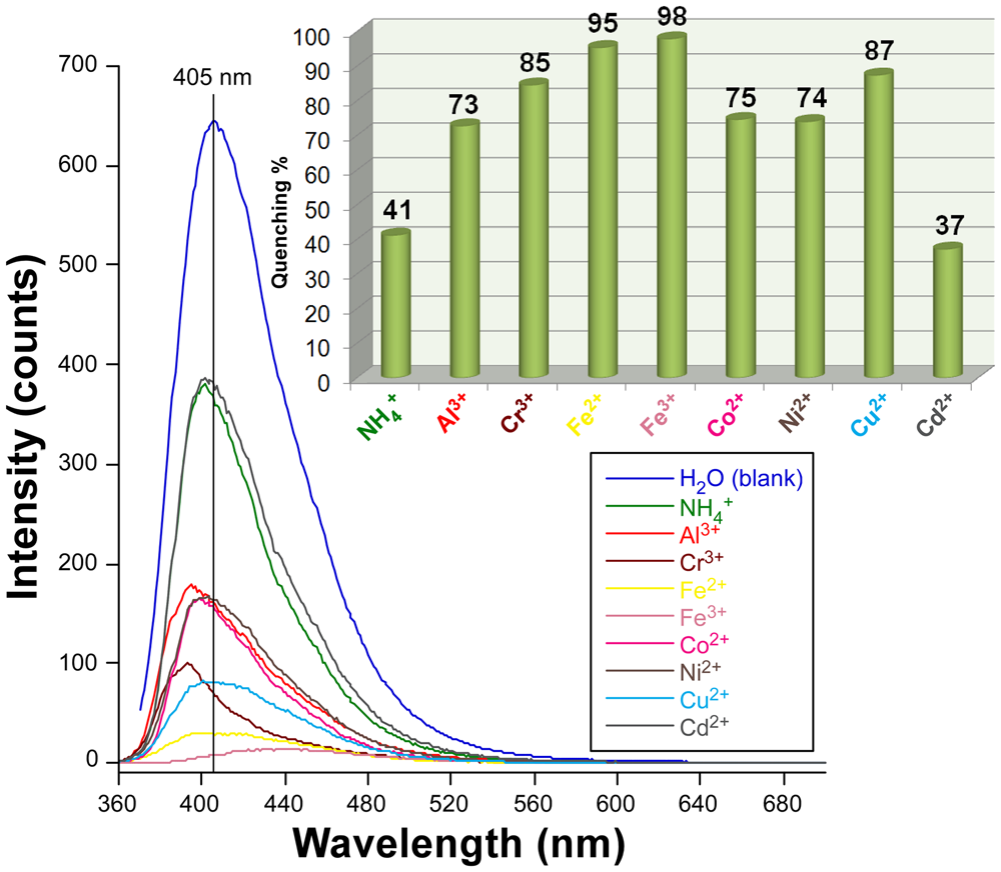
PL response of H_2_O@**3** against common metal ions (λ_ex_ = 350 nm). Inset shows the quenching percentage estimated from the maximum emission intensity respect to a blank solution.

In view of the intense PL signal observed for the H_2_O@**3** suspension (the least quenching brought by water compared to other solvents), the detection of ions in aqueous solutions was also studied. As inferred from Fig. 10, **3** presents a variable sensing capacity to ions according to their quenching capacity that follows the series: Cd^2+^ <NH_4_^+^ < Al^3+^ <Ni^2+^ <Co^2+^ <Cr^3+^ <Cu^2+^ <Fe^2+^ <Fe^3+^. It is important to notice that the quenching percentage (QP) at 10 mM is above 90% for both iron cations, among which the most stable oxidation state almost clears the PL emission. This behaviour meets the expected response given that UV-Vis absorption band of these ions overlap substantially well with the excitation band, such that most of the light irradiated does not reach the dispersed solid of **3**^32c^. More interestingly, the presence of most of studied metal ions promotes a shift of the emission band, most of which cause a blue-shift which can be as large as 15 nm (for Cr^3+^@**3**). However, the strongest quencher of the PL, Fe^3+^ ion, promotes an opposed response by red-shifting the maximum up to ca. λ_em_ = 440 nm. This overall behaviour seems to indicate that metal ions are able to interact with the ligands of the backbone.

**Figure 11:**
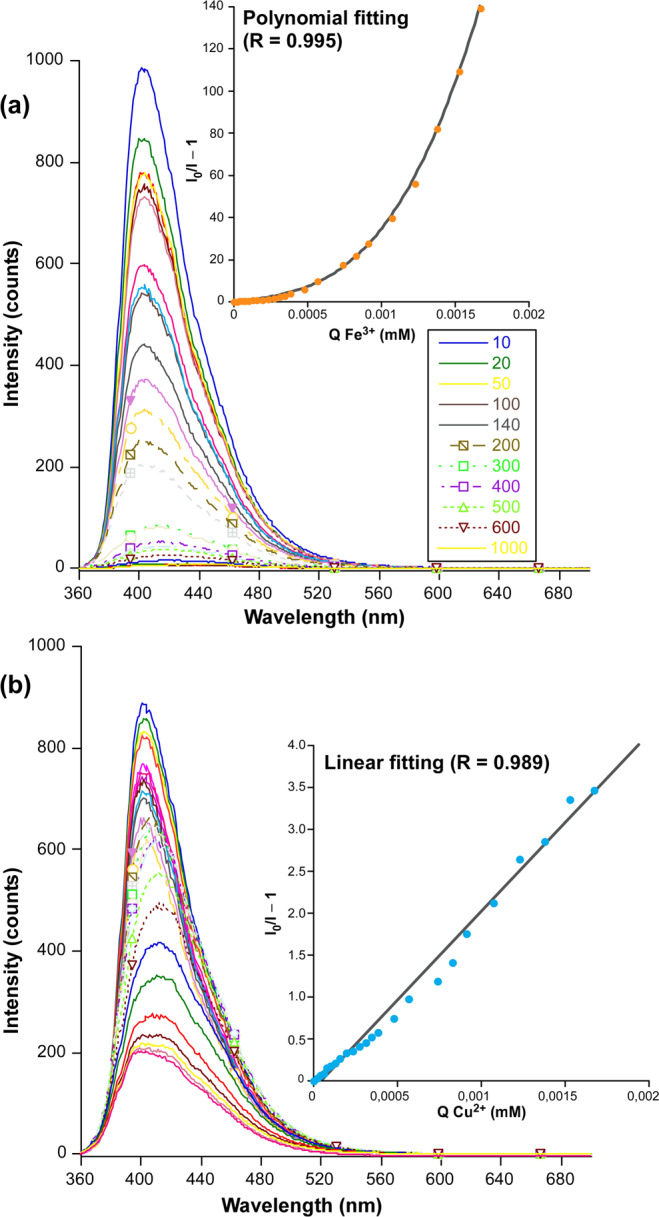
Luminescence quenching of H_2_O@**3** with gradual addition (microliters of metal ion solutions added are shown in the capture with different colours of **(a)** Fe^3+^ and **(b)** Cu^2+^ ions. Stern-Volmer plots showing the best fits are also shown.

Taking into account the previous metal-dependent turn-off results, we decided to explore the PL response of the CP for Fe^3+^ and Cu^2+^ ions, for which an exhaustive evolution of the emission intensity was monitored by gradually increasing the concentration of the metal quencher. Note that no further study was conducted for Fe^2+^ because of its spontaneous oxidation in waste waters. Stern-Volmer plot analyses for the latter two ions exhibit a distinct behaviour. The PL signal exhibits a progressive and polynomial quenching for Fe^3+^ ion from low concentrations on, which points out the coexistence of static and dynamic quenching phenomena. On its part, the quenching by Cu^2+^ ions shows a progressive and linear quenching occurring from low concentration (Q) of 4 10^–5^ M. Fitting of the evolution with the respective expression (see ESI) for both ions gives values of *K*_*sv*_ of 1.79 10^4^ and 2.12 10^4^ M^–1^ (Fig. 11) with calculated limit of detection (LOD) values of 55 μM and 162 μM for Fe^3+^ and Cu^2+^ ions, respectively. Note that, despite the relatively high LODs, the estimated *K*_*sv*_ value for Fe^3+^ detection can be considered quite promising compared to other reported MOFs with characteristic iron sensing capacity, usually exhibiting values within the 10^4^ – 10^5^ range^19,60,61^, among which the *K*_*sv*_ = 2.67 10^5^ M^–1^ reported for the MOF of {[(CH_3_)_2_NH_2_]_6_[Cd_3_L(H_2_O)_2_]·12H_2_O}_n_ formula deserves to be mentioned^62^. In this regard, despite the dominant dynamic quenching observed for these ions (in view of their large *K*_*sv*_), significant static quenching may be claimed for Fe^3+^ according to the non-linear distribution of the plot, a fact that may be explained according to the accessibility of the carboxylate oxygen atoms of 2ain ligands (which are also involved in hydrogen bonding interactions with exocyclic amino groups of neighbouring 2ain ligands) from the microchannels of the MOF. The recyclability of **3** has been also checked by PXRD measurement on the solid filtered and dried after the experiment (see ESI). Thinking on a potential use of the compound for sensing applications on polluted wastewaters, an additional analysis was accomplished in order to explore the specific response against similar quenchers, for which the mixed Fe^3+^/Cu^2+^ system was studied. In view of the obtained Stern-Volmer plot, the quenching evolution of the material is not proportional to the sum of both isolated ions, but it shows a linear response after a concentration of 1 10^-4^ M of each individual ion. Fitting of the linear part gives a *K*_*sv*_ of 2.12 10^4^ M^–1^, which allows us concluding that there is a tough competition between both quenchers to interact with the framework.

## Conclusions

Three metal-organic framework materials, of {[M(μ-2ain)_2_]·DMF}_n_ formula, based on first row transition metal ions (Co^II^, Ni^II^, and Zn^II^) and 2-aminoisonicotinate (2ain) ligand have been synthesized and chemically and structurally characterized. Their crystal structure consists of a doubly-interpenetrated three-dimensional open architecture which contains microchannels filled with solvent molecules. Taking advantage of the porosity and their magnetic and/or photoluminescence (PL) properties, their guest-dependent magnetic and PL response has been evaluated. On the one hand, the weak exchange interactions transmitted through the chiral coordination network leads to a spin-glass behaviour that governs the magnetism of the cobalt-based counterpart, though no frequency-dependent signal is observed for the nickel compound. Interestingly, when DMSO and MeOH are loaded within the pores of this MOF, exchanging the pristine DMF molecules, the crystal building undergoes a kind of rearrangement which modulates the magnetic properties of the material. In particular, the glass-like magnetic relaxation gives way to substantial SIM behaviour probably derived from the first-order SOC inherent to cobalt(II) ions, consisting of multiple spin-phonon processes characterized by low blocking temperature but relatively high energy barriers. On another level, solid state PL measurements show that the zinc-based MOF displays strong bright blue emissions arising from a LCCT mechanism (based on π–π* or n–π* transitions in 2ani) as confirmed by TD-DFT calculations. The vibrational quenching may be efficiently prevented at low temperature (10 K), since a strong gain in integrated emitted intensity (of 55 times) is observed compared to RT. This MOF exhibits clear emission dependence in contact with solvents under a constant excitation wavelength, where the emission quenching increases according to the decreasing polarity of the solvent (moving from H_2_O > solvents > THF). With regard to its sensing towards metal ions in aqueous solutions, Cu^2+^, Fe^2+^, and Fe^3+^ ions are found to quench the emission to a large extent. Stern-Volmer plots for aqueous Cu^2+^ and Fe^3+^ suspensions containing Zn-2ain reveal a similar detection capacity (*K*_*sv*_ of 1.79 10^4^ and 2.12 10^4^ M^–1^, respectively) but distinct mechanisms, confirming the capacity of these ions to interact with MOF. In fact, the analysis carried out on a mixture of both ions, Fe^3+^/Cu^2+^, shows a quenching evolution distinct to the sum of individual atoms, indicating a competing quenching of both quenchers to interact with the framework. All in all, the studies confirm that these MOFs modulate their properties according to solvent-exchange and/or capture of metal ions in liquid media, which paves the way to their use as sensors.

## Experimental Section

### Chemicals

All chemicals were of reagent grade and were used as commercially obtained.

#### Synthesis of {[Co(μ-2ain)_2_]·DMF}_n_ (**1**)

5 mL of a DMF solution containing 0.20 mmol of Co(NO_3_)_2_·6H_2_O (0.0582 g) were added dropwise under continuous stirring over 15 mL of a DMF/H_2_O (1:1) solution of H2ain ligand (0.40 mmol, 0.0552 g) at 70 °C. The final pH of the solution was 5.6. The dark pink coloured solution was introduced in a Teflon-lined container enclosed into a stainless steel autoclave solution, where it was heated up to 120 °C for two days. The mixture was slowly cooled down to room temperature and purple plate shaped single crystals were observed when opening the recipient. Crystals were filtered off and washed several times with water and methanol. Yield 50–60% (based on metal). Homogeneity and purity of samples were checked by means of elemental and thermogravimetric analyses, FT-IR, and X-ray powder diffraction data (see section S5 in the ESI). Anal. Calcd. for (hydrated) C_15_H_19_CoN_5_O_6_ (%): C, 44.35; H, 4.22; Co, 14.51; N, 17.24. Found: C, 42.32; H, 4.38; Co, 13.83; N, 16.60.

#### Synthesis of {[Ni(μ-2ain)_2_]·DMF}_n_ (**2**)

Well shaped single crystals of compound **2** were collected from a Teflon-lined vessel after carrying out the same experimental procedure reported for **1** but for the use of Ni(NO_3_)_2_·6H_2_O (0.0582 g) instead of the cobalt source. Yield of 45–50% (based on metal). Anal. Calcd. for (hydrated) C_15_H_19_N_5_NiO_6_ (%): C, 44.37; H, 4.22; N, 17.25; Ni, 14.45. Found: C, 42.65; H, 4.32; N, 16.45; Ni, 13.74.

#### Synthesis of {[Zn(μ-2ain)_2_]·DMF}_n_ (**3**)

Following the same above mentioned synthetic conditions with Zn(NO_3_)_2_·6H_2_O (0.0595 g) gave rise to the growth of colourless single crystals of **3**. Yield of 50–55% (based on metal). Anal. Calcd. for (hydrated) C_15_H_19_N_5_O_6_Zn (%): C, 43.65; H, 4.15; N, 16.97; Zn, 15.84. Found: C, 43.25; H, 4.34; N, 16.41; Zn, 15.02.

#### Physical measurements

Elemental analyses (C, H, N) were performed on an Euro EA Elemental Analyzer and the metal content determined by inductively coupled plasma (ICP-AES) was performed on a Horiba Yobin Yvon Activa spectrometer. IR spectra (KBr pellets) were recorded on a ThermoNicolet IR 200 spectrometer in the 4000 − 400 cm^−1^ spectral region. Magnetic susceptibility measurements were performed on polycrystalline samples of the complexes with a Quantum Design SQUID MPMS-7T susceptometer at an applied magnetic field of 1000 G. The susceptibility data were corrected for the diamagnetism estimated from Pascal’s Tables^63^, the temperature-independent paramagnetism, and the magnetization of the sample holder. *Ac* measurements were performed on a Physical Property Measurement System-Quantum Design model 6000 magnetometer under a 3.5 G *ac* field and frequencies ranging from 60 to 10 000 Hz. Thermal analyses (TG/DTA) were performed on Mettler-Toledo TGA/SDTA851 thermal analyser in a synthetic air atmosphere (79% N_2_ / 21% O_2_) with a heating rate of 5 °C·min^–1^. A closed cycle helium cryostat enclosed in an Edinburgh Instruments FLS920 spectrometer was employed for steady state photoluminescence (PL) and lifetime measurements in the 10 − 300 K range. All samples are first placed under high vacuum (of *ca*. 10^−9^ mbar) to avoid the presence of oxygen or water in the sample holder. For steady-state measurements a Müller-Elektronik-Optik SVX1450 Xe lamp or an IK3552R-G He-Cd continuous laser (325 nm) were used as excitation source, whereas a microsecond pulsed lamp was employed for recording the lifetime measurements. Photographs of irradiated single-crystal and polycrystalline samples were taken at room temperature in a micro-PL system included in an Olympus optical microscope illuminated with a Hg lamp. The PL quantum yield was measured in solid state using a Horiba Quanta-ϕ F-3029 integrating sphere.

#### X-ray Diffraction Data Collection and Structure Determination

X-ray data collection of suitable single crystals was done at 100(2) K on a Bruker VENTURE area detector equipped with graphite monochromated Mo K_α_ radiation (λ = 0.71073 Å) by applying the ω-scan method. Data reduction were performed with the APEX2^64^ software and corrected for absorption using SADABS^65^. Crystal structures were solved by direct methods using the SIR97 program^66^ and refined by full-matrix least-squares on F^2^ including all reflections employing the WINGX crystallographic package^67,68^. All hydrogen atoms were located in difference Fourier maps and included as fixed contributions riding on attached atoms with isotropic thermal displacement parameters 1.2 times or 1.5 times those of their parent atoms for the organic ligands and the water molecules, respectively. Lattice solvent molecules placed in the voids of the structures found to be highly disordered due to the high symmetry acquired by the framework. Therefore, the final refinement was made with an hkl file provided by the SQUEEZE routine implemented in PLATON^38^, which removed the latter electron density. Details of the structure determination and refinement of all compounds are summarized in Table 4. Crystallographic data for the crystal structures have been deposited with the Cambridge Crystallographic Data Center as supplementary publication nos. CCDC 1942731-1942733. Copies of the data can be obtained free of charge on application to the Director, CCDC, 12 Union Road, Cambridge, CB2 1EZ, U.K. (Fax: +44-1223-335033; e-mail: deposit@ccdc.cam.ac.uk or http://www.ccdc.cam.ac.uk).

**Table 4:** Single crystal X-ray diffraction data and structure refinement details of compounds **1**, **2** and **3**.

	1	2	3
Empirical formula	C_15_H_17_CoN_5_O_5_	C_15_H_17_N_5_NiO_5_	C_15_H_17_N_5_O_5_Zn
Formula weight	424.28	424.04	430.73
Crystal system	orthorhombic	orthorhombic	orthorhombic
Space group	*Fddd*	*Fddd*	*Fddd*
*a* (Å)	12.693(1)	12.649(1)	12.903(1)
*b* (Å)	22.776(2)	22.289(2)	22.869(2)
*c* (Å)	23.847(2)	23.949(2)	23.890(2)
V (Å^3^)	6894(1)	6752(1)	7049(1)
Z	16	16	16
Reflections collected	19730	10466	10922
Unique/parameters	2112/96	1998/96	2121/96
Rint	0.0597	0.0323	0.0314
GoF (S)^a^	1.038	1.058	1.077
R_1_^b^/wR^2c^ [I > 2σ(I)]	0.0344/0.0840	0.0282/0.0746	0.0303/0.0801
R_1_^b^/wR^2c^ [all]	0.0462/0.0900	0.0318/0.0771	0.0367/0.0835

[a]S = [∑w(F_0_^2^ – F_c_^2^)^2^ / (N_obs_ – N_param_)]^1/2^ [b] R_1_ = ∑ | | F_0_ | – | F_c_ | |/∑|F_0_ | [c] wR_2_ = [∑w(F_0_^2^ – F_c_^2^)^2^/∑wF_0_^4^]^1/2^; w = 1/[σ^2^(F_0_^2^) + (aP)^2^ + bP] where P = (max(F_0_^2^,0) + 2Fc^2^)/3 with a = 0.0472 (**1**), 0.0416 (**2**), 0.0459 (**3**); and b = 8.1485 (**1**), 10.0330 (**2**), and 1.3767 (**3**).

The X-ray powder diffraction (XRPD) patterns were collected on a Phillips X’PERT powder diffractometer with Cu-K_α_ radiation (λ = 1.5418 Å) over the range 5 < 2θ < 50° with a step size of 0.026° and an acquisition time of 2.5 s per step at 25 °C. Indexation of the diffraction profiles were made by means of the FULLPROF program (pattern-matching analysis)^69^ on the basis of the space group and the cell parameters found by single crystal X-ray diffraction. The unit cell parameters obtained in the final refinement are listed in the Supporting Information.

#### Computational details

The computational strategy adopted in this work to compute the magnetic coupling constant (*J*_*calc*_) values has been described and validated elsewhere^70^. One calculation was performed to determine the high-spin state and another to determine the low-spin broken symmetry state. The correctness of the latter state was ensured by means of its spin density distribution. Density functional theory was used to perform two separate calculations to evaluate the coupling constant of each compound, employing the aforementioned hybrid B3LYP functional and Gaussian-implemented 6-311 G(d) basis set for all non-metal atoms and the corresponding LANL2DZ pseudopotentials for the metal atoms. Spin-density surfaces were plotted using GaussView 5^71^.

PL spectra were calculated by means of TD-DFT using the Gaussian 09 package^72^, using the Becke three parameter hybrid functional with the non-local correlation functional of Lee-Yang-Parr (B3LYP)^73,74^ along with 6-311 G + + (d,p) basis set^75^ was adopted for all atoms but for the central zinc cation, for which the LANL2DZ^76^ basis set along with the corresponding effective core potential (ECP) was used. The 40 lowest excitation states were calculated by the TD-DFT method. Results were analysed with GaussSum program package^77^ and molecular orbitals plotted using GaussView 5.

## Supplementary information

Supplementary information.

checkcif_Compound1.pdf

checkcif_Compound2.pdf

checkcif_Compound1.pdf

Compound1.cif

Compound2.cif

Compound3.cif

## References

[1] Kreno LE (2012). Metal–Organic Framework Materials as Chemical Sensors. Chem. Rev..

[2] Cohen SM (2012). Postsynthetic Methods for the Functionalization of Metal–Organic Frameworks. Chem. Rev..

[3] Falcaro P (2014). MOF positioning technology and device fabrication. Chem. Soc. Rev..

[4] Meek ST, Greathouse JA, Allendorf MD (2011). Metal‐Organic Frameworks: A Rapidly Growing Class of Versatile Nanoporous Materials. Adv. Mater..

[5] Cheetham AK, Rao CNR (2007). Materials science. There’s room in the middle. Science.

[6] Tranchemontagne DJ, Mendoza-Cortés JL, O’Keeffe M, Yaghi OM (2009). Secondary building units, nets and bonding in the chemistry of metal–organic frameworks. Chem. Soc. Rev..

[7] Cepeda J, Beobide G, Castillo O, Luque A, Pérez-Yáñez S (2017). Structural diversity of coordination compounds derived from double-chelating and planar diazinedicarboxylate ligands. Coord. Chem. Rev..

[8] Eddaoudi M, Sava DF, Eubank JF, Adil K, Guillerm V (2015). Zeolite-like metal–organic frameworks (ZMOFs): design, synthesis, and properties. Chem. Soc. Rev..

[9] Janiak CH, Vieth JK (2010). MOFs, MILs and more: concepts, properties and applications for porous coordination networks (PCNs). New J. Chem..

[10] Coronado E, Dumbar KR (2009). Inorg. Chem..

[11] Li J-R, Sculley J, Zhou H-C (2012). Metal–Organic Frameworks for Separations. Chem. Rev..

[12] Cepeda J (2018). Modulation of pore shape and adsorption selectivity by ligand functionalization in a series of “rob”-like flexible metal–organic frameworks. J. Mater. Chem. A.

[13] Dhakshinamoorthy A, Garcia H (2014). Metal–organic frameworks as solid catalysts for the synthesis of nitrogen-containing heterocycles. Chem. Soc. Rev..

[14] Ma L, Abney C, Lin W (2009). Enantioselective catalysis with homochiral metal–organic frameworks. Chem. Soc. Rev..

[15] Horcajada P (2012). Metal–Organic Frameworks in Biomedicine. Chem. Rev..

[16] Lustig WP (2017). Metal-organic frameworks: functional luminescent and photonic materials for sensing applications. Chem. Soc. Rev..

[17] Kozlowski H (2009). Copper, iron, and zinc ions homeostasis and their role in neurodegenerative disorders (metal uptake, transport, distribution and regulation). Coord. Chem. Rev..

[18] Mølhave L, Bach B, Pedersen OF (1986). Human reactions to low concentrations of volatile organic compounds. Environ. Int..

[19] Yi F-Y, Chen D, Wu M-K, Han L, Jiang H-L (2016). Chemical Sensors Based on Metal–Organic Frameworks. ChemPlusChem.

[20] Guo H, Lee SC, Chan LY, Li WM (2004). Risk assessment of exposure to volatile organic compounds in different indoor environments. Environ. Res..

[21] You L, Zha D, Anslyn EV (2015). Recent Advances in Supramolecular Analytical Chemistry Using Optical Sensing. Chem. Rev..

[22] Allendorf MD, Bauer CA, Bhaktaa RK, Houk RJT (2009). Luminescent metal–organic frameworks. Chem. Soc. Rev..

[23] Lower SK, El-Sayed MA (1966). The Triplet State and Molecular Electronic Processes in Organic Molecules. Chem. Rev..

[24] Cepeda J, Rodríguez-Diéguez A (2016). Tuning the luminescence performance of metal–organic frameworks based on d10 metal ions: from an inherent versatile behaviour to their response to external stimuli. CrystEngComm.

[25] Mínguez Espallargas G, Coronado E (2018). Magnetic functionalities in MOFs: from the framework to the pore. Chem. Soc. Rev..

[26] Frost JM, M. Harriman KL, Murugesu M (2016). The rise of 3-d single-ion magnets in molecular magnetism: towards materials from molecules?. Chem. Sci.

[27] Craig GA, Murrie M (2015). 3d single-ion magnets. Chem. Soc. Rev..

[28] Seco JM, Oyarzabal I, Pérez-Yáñez S, Cepeda J, Rodríguez-Diéguez A (2016). Designing Multifunctional 5-Cyanoisophthalate-Based Coordination Polymers as Single-Molecule Magnets, Adsorbents, and Luminescent Materials. Inorg. Chem..

[29] Murrie M (2010). Cobalt(II) single-molecule magnets. Chem. Soc. Rev..

[30] Gómez-Coca S, Aravena D (2015). Morales, & Ruiz, E. Large magnetic anisotropy in mononuclear metal complexes. Coord. Chem. Rev..

[31] Chen L (2014). Slow Magnetic Relaxation in a Mononuclear Eight-Coordinate Cobalt(II) Complex. J. Am. Chem. Soc..

[32] Cepeda J (2016). A Zn based coordination polymer exhibiting long-lasting phosphorescence. Chem. Commun..

[33] Pajuelo-Corral O (2019). Alkaline-earth and aminonicotinate based coordination polymers with combined fluorescence/long-lasting phosphorescence and metal ion sensing response. J. Mater. Chem. C.

[34] Rodríguez-Diéguez A, Pérez-Yáñez S, Ruiz-Rubio L, Seco JM, Cepeda J (2017). From isolated to 2D coordination polymers based on 6-aminonicotinate and 3d-metal ions: towards field-induced single-ion-magnets. CrystEngComm.

[35] Pachfule P, Chen Y, Jiang J, Banerjee R (2011). Experimental and computational approach of understanding the gas adsorption in amino functionalized interpenetrated metal organic frameworks (MOFs). J. Mater. Chem..

[36] TOPOS Main Page, http://www.topos.ssu.samara.ru (accessed February 14, 2019).

[37] Blatov VA, Shevchenko AP, Proserpio DM (2014). Applied Topological Analysis of Crystal Structures with the Program Package. Cryst. Growth Des..

[38] Spek AL (2009). Structure validation in chemical crystallography. Acta Cryst.

[39] Lloret F, Julve M, Cano J, Ruiz-Garcia R, Pardo E (2008). Magnetic properties of six-coordinated high-spin cobalt(II) complexes: Theoretical background and its application. Inorg. Chim. Acta.

[40] Sakiyama Hiroshi, Ito Rie, Kumagai Hitoshi, Inoue Katsuya, Sakamoto Masatomi, Nishida Yuzo, Yamasaki Mikio (2001). Dinuclear Cobalt(II) Complexes of an Acyclic Phenol-Based Dinucleating Ligand with Four Methoxyethyl Chelating Arms − First Magnetic Analyses in an Axially Distorted Octahedral Field. European Journal of Inorganic Chemistry.

[41] Rueff, J.-M., Masciocchi, N., Rabu, P., Sironi, A. & Skoulios, A. Structure and Magnetism of a Polycrystalline Transition Metal Soap − CoII[OOC(CH2)10COO](H2O)2. *Eur. J. Inorg. Chem*. 2843–2848 (2001).

[42] Cui L (2014). Solvents and auxiliary ligands co-regulate three antiferromagnetic Co(II) MOFs based on a semi-rigid carboxylate ligand. Dalton Trans..

[43] Lloret F, Julve M, Cano J, Ruiz-García R, Pardo E (2008). Magnetic properties of six-coordinated high-spin cobalt(II) complexes: Theoretical background and its application. Inorg. Chim. Acta.

[44] Chilton NF, Anderson RP, Turner LD, Soncinia A, Murray KS (2013). PHI: a powerful new program for the analysis of anisotropic monomeric and exchange-coupled polynuclear d- and f-block complexes. J. Comput. Chem..

[45] Boča R (2004). Zero-field splitting in metal complexes. Coord. Chem. Rev..

[46] Gómez-Coca S (2014). Origin of slow magnetic relaxation in Kramers ions with non-uniaxial anisotropy. Nat. Commun..

[47] Yao P-F (2018). Hierarchical Assembly of a {Co24} Cluster from Two Vertex-Fused {Co13} Clusters and Their Single-Molecule Magnetism. Inorg. Chem..

[48] Liu Y, Shi W-J, Lu Y-K, Liu G, Hou L (2019). & Wang, Y.-Y. Nonenzymatic Glucose Sensing and Magnetic Property Based On the Composite Formed by Encapsulating Ag Nanoparticles in Cluster-Based Co-MOF. Inorg. Chem..

[49] Du Z-Y (2017). Two magnetic Δ-chain-based Mn(II) and Co(II) coordination polymers with mixed carboxylate-phosphinate and μ_3_-OH^−^ bridges. CrystEngComm.

[50] Zhang X-M, Li P, Gao W, Liu J-P, Gao E-Q (2015). 3D Co(II) coordination polymer with ferromagnetic-like layers based on azide and tetrazolate bridges showing slow magnetic dynamics. Dalton Trans..

[51] García-Valdivia AA, Seco JM, Cepeda J, Rodríguez-Diéguez A (2017). Designing Single-Ion Magnets and Phosphorescent Materials with 1-Methylimidazole-5-carboxylate and Transition-Metal Ions. Inorg. Chem..

[52] Herchel R, Váhovská L, Potocnak I, Travnicek Z (2014). Slow Magnetic Relaxation in Octahedral Cobalt(II) Field-Induced Single-Ion Magnet with Positive Axial and Large Rhombic Anisotropy. Inorg. Chem..

[53] Nyokong T (2007). Effects of substituents on the photochemical and photophysical properties of main group metal phthalocyanines. Coord. Chem. Rev..

[54] San Sebastian, E., Rodríguez-Diéguez, A., Seco, J. M. & Cepeda, J. Coordination Polymers with Intriguing Photoluminescence Behavior: The Promising Avenue for Greatest Long-Lasting Phosphors. *Eur. J. Inorg. Chem*., 2155–2174 (2018).

[55] A. Beeby *et al*. *Chem. Soc., Perkin Trans*. **2**, 493–503 (1999).

[56] Melavanki RM, Kusanur RA, Dadadevaramath JS, Kulkarni MV (2010). Effect of solvent polarity on the fluorescence quenching of biologically active 5BAMC by aniline in binary solvent mixtures. J. Fluores.

[57] Ma D (2010). n situ2,5-pyrazinedicarboxylate and oxalate ligands synthesis leading to a microporous europium–organic framework capable of selective sensing of small molecules. CrystEngComm.

[58] Qiu, Y. C. *et al*. In situtetrazoleligand synthesis leading to a microporous cadmium–organic framework for selective ion sensing. *Chem. Commun*. 5415–5417 (2009). 10.1039/b907783a 19724803

[59] Liu X-J (2016). A Water-Stable Metal−Organic Framework with a Double-Helical Structure for Fluorescent Sensing. Inorg. Chem..

[60] He J, Xu J, Yin J, Li N, Bu X-H (2019). Recent advances in luminescent metal-organic frameworks for chemical sensors. Sci China Mater.

[61] Gao Q, Xu J, Bu X-H (2019). Recent advances about metal–organic frameworks in the removal of pollutants from wastewater. Coord. Chem. Rev..

[62] Lu BB, Jiang W, Yang J, Liu YY, Ma JF (2017). Resorcin[4]arene-based Microporous Metal-Organic Framework as an Efficient Catalyst for CO_2_ Cycloaddition with Epoxides and Highly Selective Luminescent Sensing of Cr_2_O_7_^2-^. ACS Appl. Mater. Interfaces.

[63] Earnshaw, A. In: *Introduction to Magnetochemistry*, Academic Press: London, 1968.

[64] Bruker. Apex2, Bruker AXS Inc: Madison, WI, 2004.

[65] Sheldrick, G. M. *SADABS, Program for Empirical Adsorption Correction*, Institute for Inorganic Chemistry, University of Gottingen: Germany, 1996.

[66] Altomare A (1999). SIR97: a new tool for crystal structure determination and refinement. J. Appl. Crystallogr.

[67] Sheldrick, G. M. *SHELX-2014, Program for Crystal Structure Refinement*, University of Göttingen: Göttingen, Germany, 2014.

[68] Farrugia LJ (1999). WinGX suite for small-molecule single-crystal crystallography. J. Appl. Crystallogr.

[69] Rodríguez-Carvajal, J. *FULLPROF, version July-2017*, Laboratoire Léon Brillouin (CEA-CNRS), Centre d'Études de Saclay, Gif sur Yvette Cedex: France, 2017.

[70] Ruiz E, Rodríguez-Fortea A, Cano J, Álvarez S, Alemany P (2003). About the calculation of exchange coupling constants in polynuclear transition metal complexes. J. Comput. Chem..

[71] Dennington, R., Keith, T., Millam, J. *GaussView*, Version 5, Semichem Inc: Shawnee Mission, KS, 2009.

[72] Frisch, M. J. *et al*. Gaussian 09, revision A.02, Gaussian, Inc., Walling-ford, CT, 2009.

[73] Becke AD (1993). A new mixing of Hartree–Fock and local density‐functional theories. J. Chem. Phys..

[74] Lee C, Yang W, Parr RG (1988). Development of the Colle-Salvetti correlation-energy formula into a functional of the electron density. Phys. Rev. B.

[75] Noodleman L, Case DA, Aizman A (1988). Broken symmetry analysis of spin coupling in iron-sulfur clusters. J. Am. Chem. Soc..

[76] Hay PJ, Wadt WR (1985). Ab initio effective core potentials for molecular calculations. Potentials for the transition metal atoms Sc to Hg. J. Chem. Phys..

[77] O’Boyle NM, Tenderholt AL, Langner K (2008). M. cclib: a library for package-independent computational chemistry algorithms. J. Comput. Chem..

