# Supplementary material for: Magnetic and Photoluminescent Sensors Based on Metal-Organic Frameworks Built up from 2-aminoisonicotinate
Source: Sci Rep. 2020 Jun 1;10:8843. doi: 10.1038/s41598-020-65687-6 (PMC7264304; doi:10.1038/s41598-020-65687-6)
Supplement: Supplementary file 1 — Supplementary information. [file 41598_2020_65687_MOESM1_ESM.pdf]

Supporting Information for:

Magnetic and Photoluminescent Sensors Based on Metal-Organic Frameworks Built up from 2-aminoisonicotinate

Antonio A. García-Valdivia,[a] Sonia Pérez-Yáñez,[b] Jose Angel García,[c] Belén Fernández,[d] Javier Cepeda*[e] and Antonio Rodríguez-Diéguez*[a]

[a]Departamento de Química Inorgánica, Facultad de Ciencias, University of Granada, 18071 Granada, Spain. [b] Departamento de Química Inorgánica, Facultad de Farmacia, Universidad del País Vasco (UPV/EHU), 01006Vitoria, Spain. [c]Departamento de Física Aplicada II, Facultad de Ciencia y Tecnología, Universidad del País Vasco/Euskal Herriko Unibertsitatea (UPV/EHU), 48940, Leioa, Spain.[d] Institute of Parasitology and Biomedicine "López-Neyra", CSIC, Av. Conocimiento s/n, 18600, Granada, Spain. [e] Departamento de Química Aplicada, Facultad de Química, Universidad del País Vasco (UPV/EHU), 20080 Donostia, Spain.

Contents:

S1. Additional figures of compounds.

S2. Additional structural data.

S3. Powder X-ray Data Collection and Analysis.

S4. Characterization of the void content of the MOFs.

S5. Thermogravimetric analysis.

S6. FT-IR spectroscopy.

S7. *Dc* magnetic susceptibility measurements.

S8. *Ac* magnetic susceptibility measurements.

S9. Spin densities of broken symmetry calculations.

S10. PL measurements of compound **3** on solid state.

S11. PL measurements on compound **3**.

S1. Additional figures and structural data of compounds.

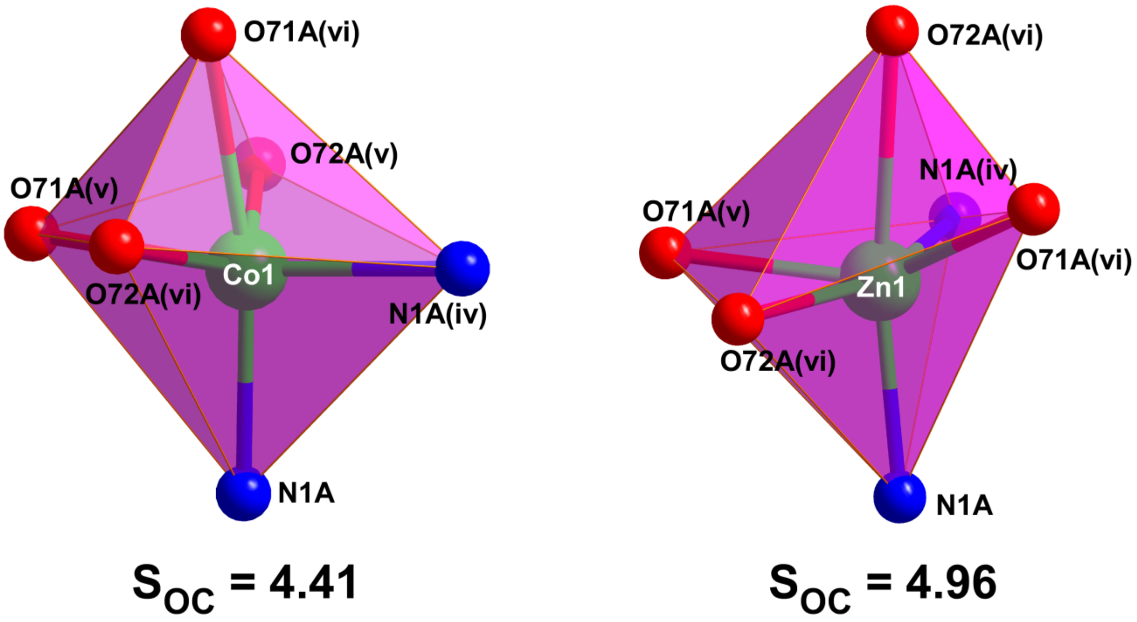

Figure S1. Coordination polyhedra of compounds **1** and **3**.

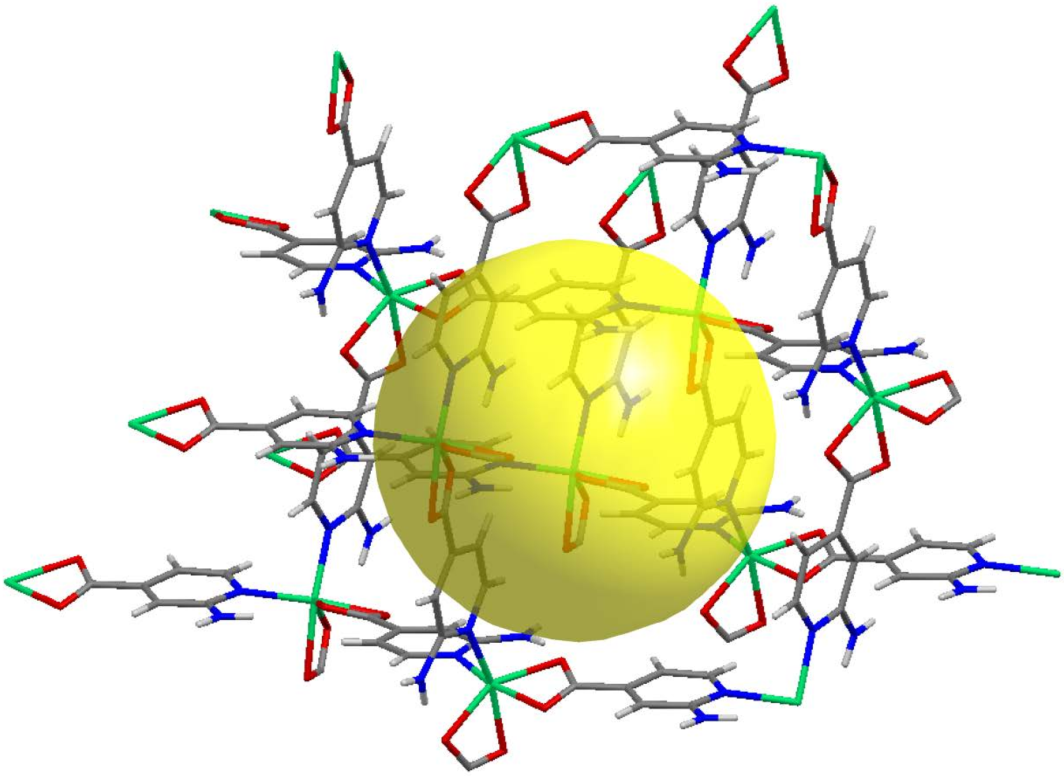

Figure S2. Fragment of the structure of compound **2**showing the potential void (sphere of ca. 9 Å diameter) left by the crystallization of one subnet.

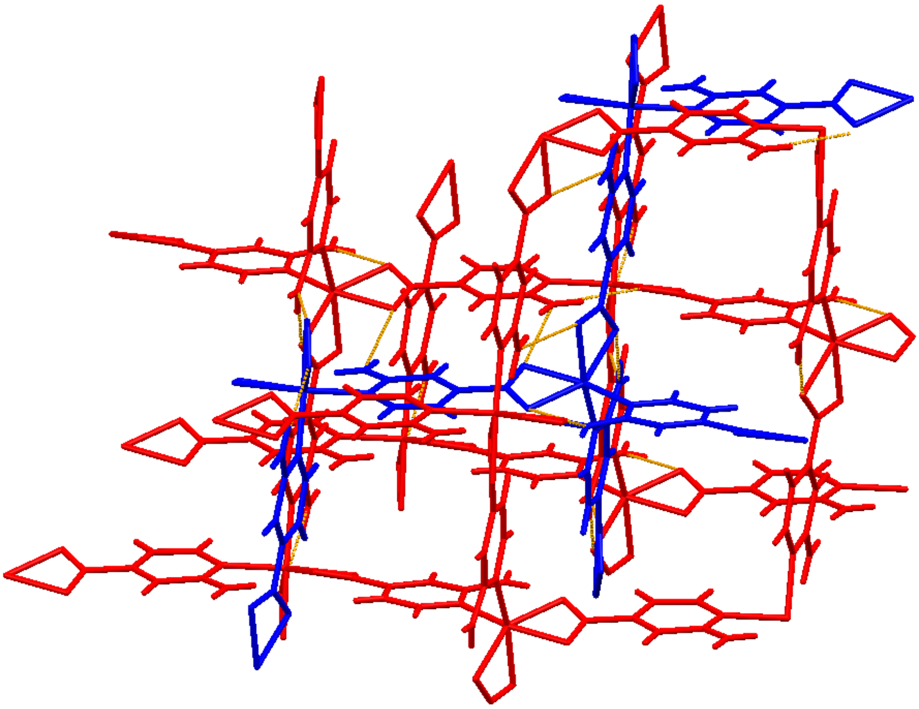

Figure S3. Interpenetration found in compound **2** showing the hydrogen bonding interactions (dashed orange lines) sustaining the overall framework.

Table S1. Structural parameters (Å, º) of hydrogen bonds (Å, º) in compounds**1–3**.[a]

D–H···A[b]	*D–H*	*H···A*	*D···A*	*D–H···A*
		Compound **1**		
N8A–H81A···O72A(vii)	0.86	2.13	2.963(2)	162.2
N8A–H81A···O71A(viii)	0.86	2.19	2.948(2)	146.9
		Compound **2**		
N8A–H81A···O71A(ix)	0.86	2.08	2.908(2)	161.2
N8A–H81A···O72A(x)	0.86	2.20	2.973(2)	148.9
		Compound **3**		
N8A–H81A···O72A(xi)	0.86	2.18	2.961(2)	151.6
N8A–H81A···O72A(xii)	0.86	2.20	3.026(2)	161.6

[a]Symmetry codes: (vii) –x, y + 1/4, z + 1/4; (viii) –x – 1/2, –y + 1/2, –z; (ix) –x –1/2, y + 1/4, z – 1/4; (x) –x + 1/2, –y + 1/2, –z; (xi) –x + 1, y + 1/4, z + 1/4. [b]D: donor. A: acceptor.

S3. Pattern-matching analyses of Powder X-ray diffraction Data.

Pattern-matching analyses confirm the purity of the polycrystalline samples.

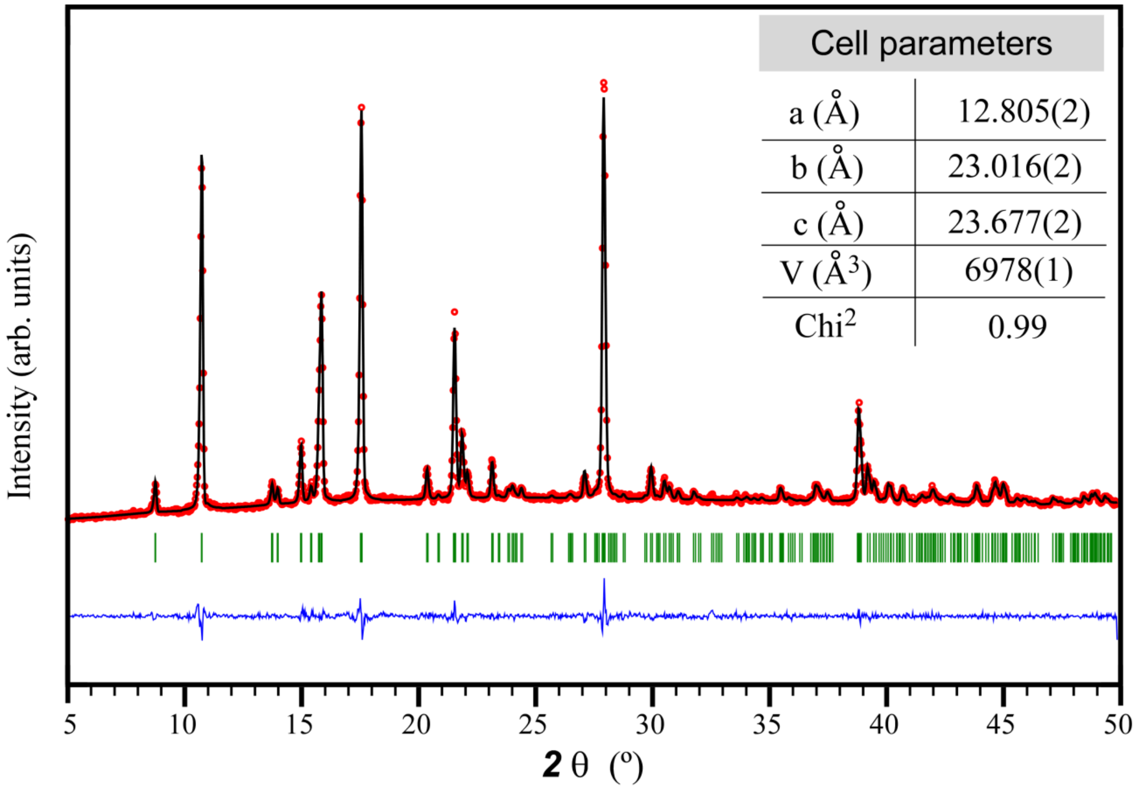

Figure S4. Full profile pattern-matching analysis of **1**.

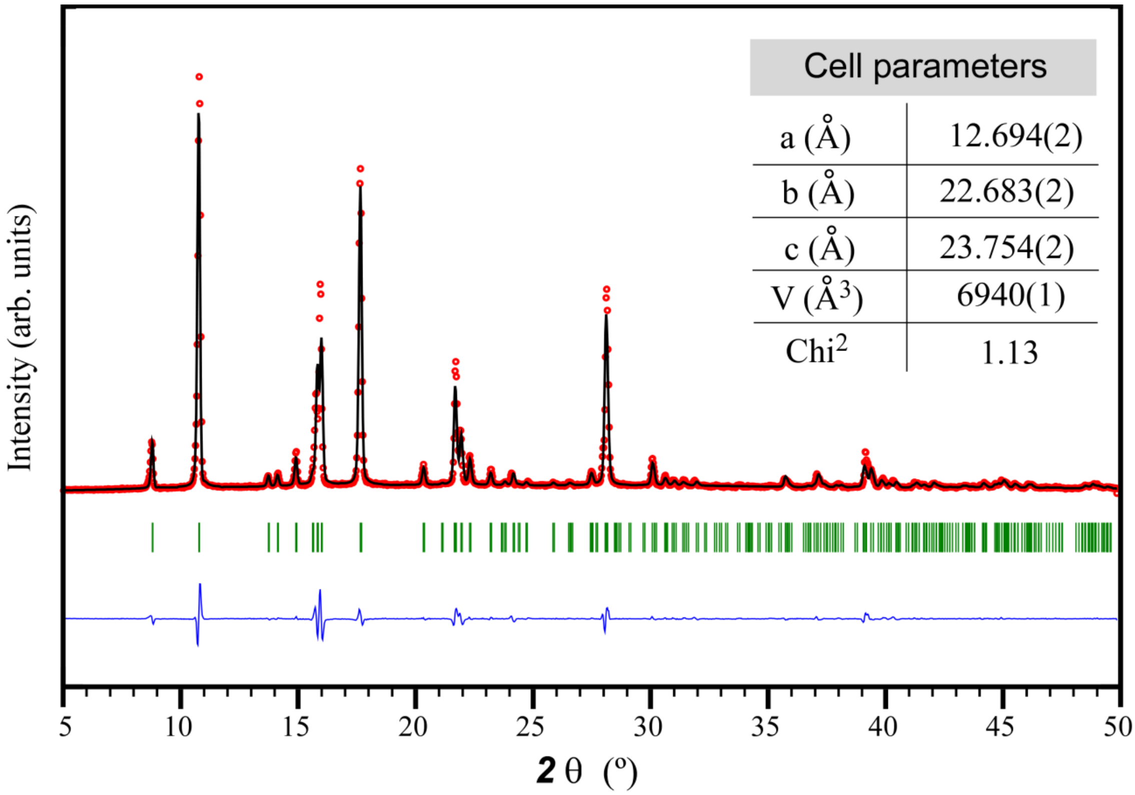

Figure S5. Full profile pattern-matching analysis of **2**.

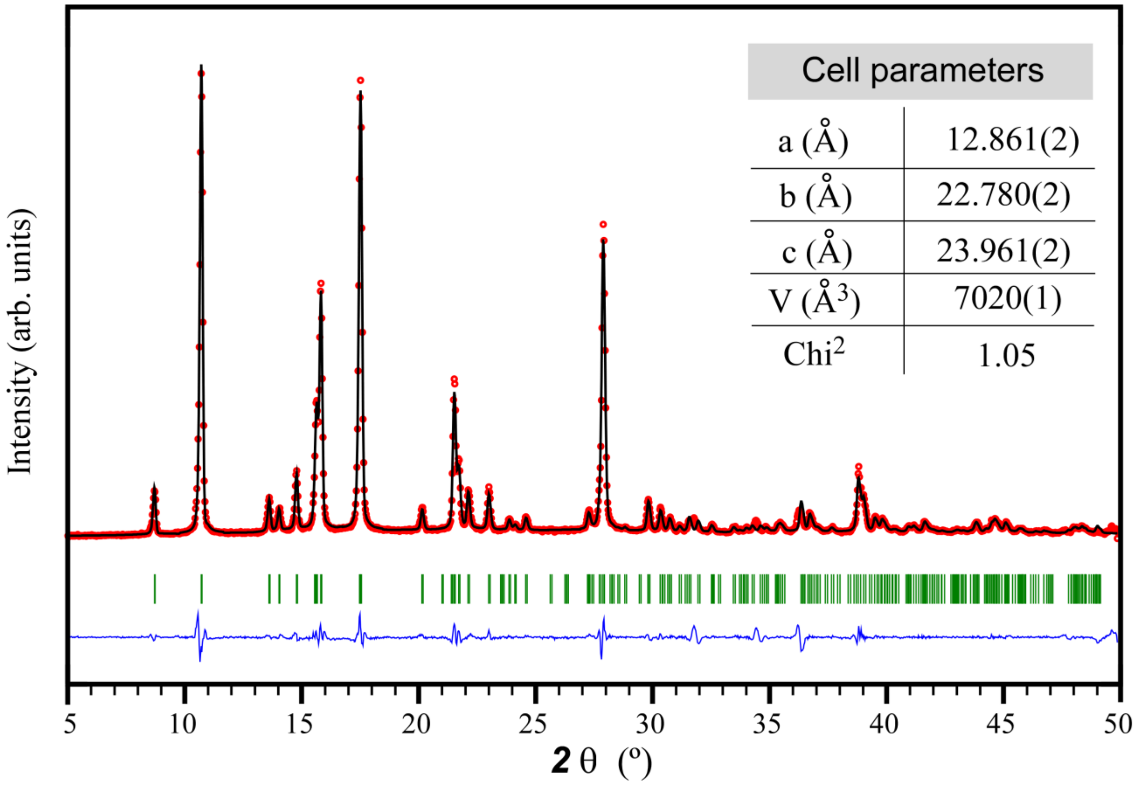

Figure S6. Full profile pattern-matching analysis of **3**.

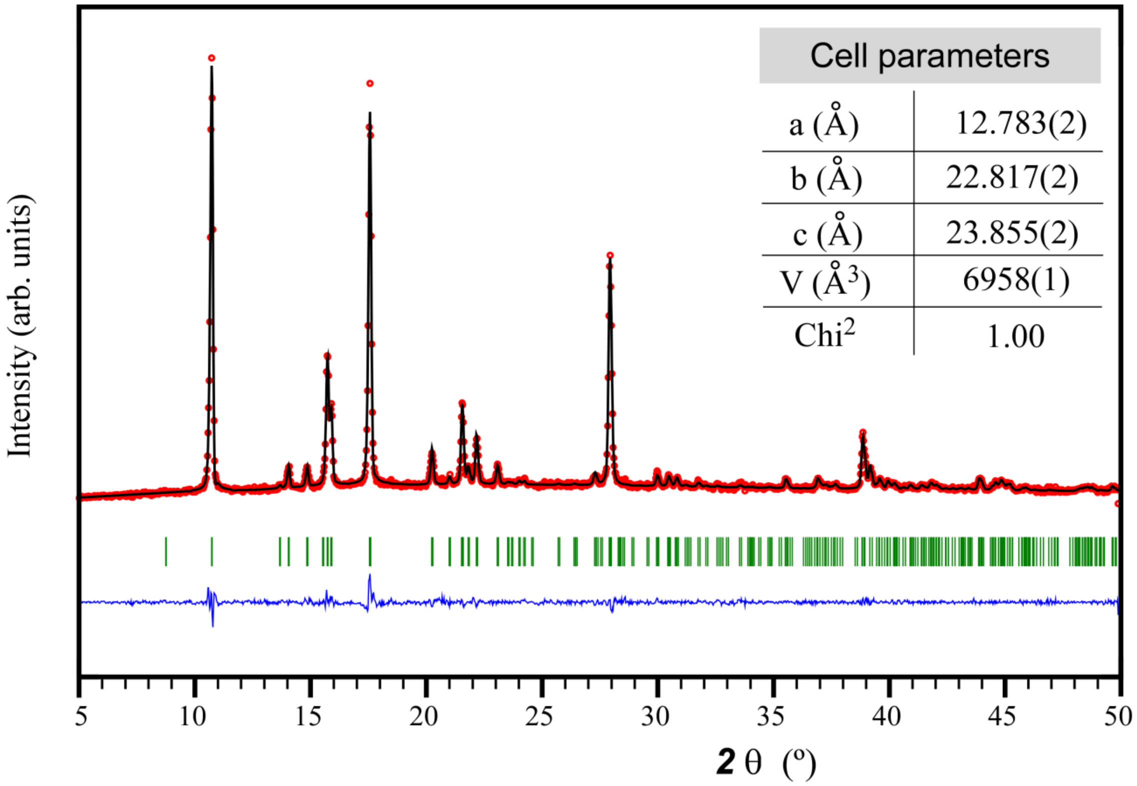

Figure S7. Full profile pattern-matching analysis of **1-DMSO**.

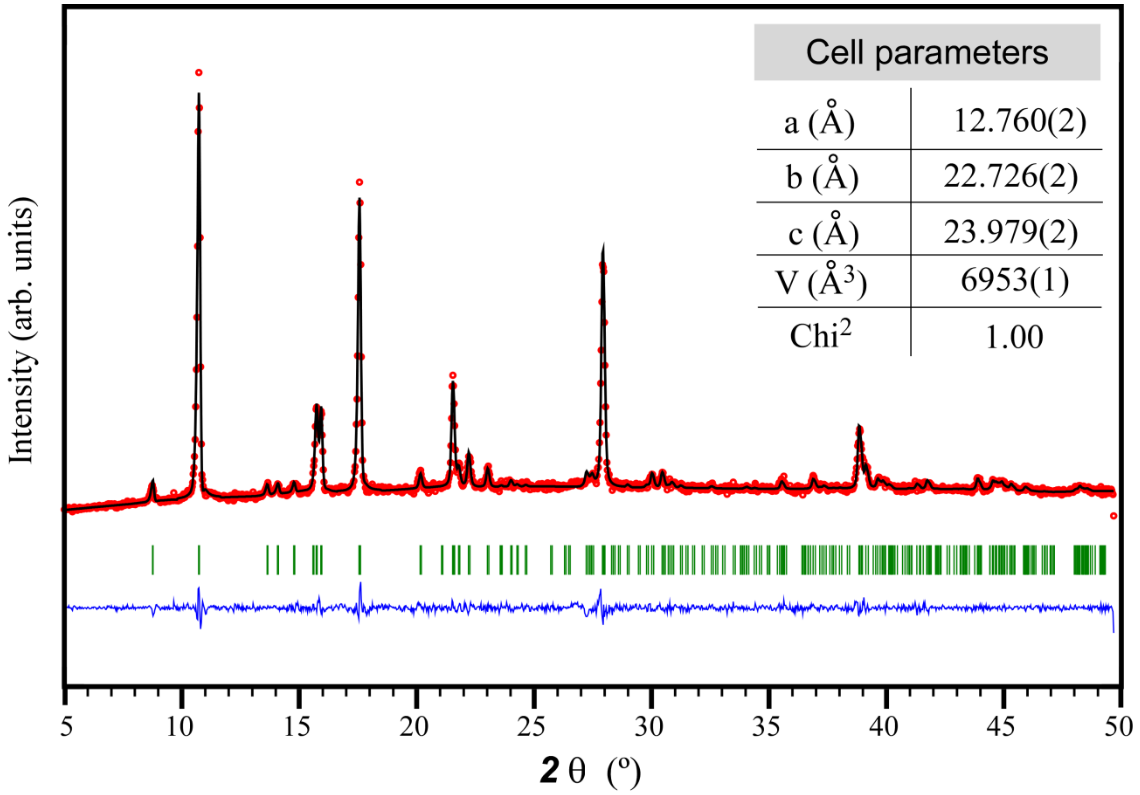

Figure S8. Full profile pattern-matching analysis of **1-MeOH**.

S4. Characterization of the void content of the MOFs.

These MOFs contains lattice solvent molecules that crystallize in a highly disordered arrangement, which prevents one determining the exact content during structure refinement stage. Accordingly, final refinement was performed with SQUEEZE routine and used to calculate the void space and the electron count. According to these results, and the fact that DMF was used as solvent without distillation, it was estimated that unit cell contain of the MOF contain voids of ca. 2500 Å^3 (2460, 2431 and 2551 Å^3 for compounds **1**, **2** and **3**) and ca. 550 electrons (542, 523 and 576 Å^3 for compounds **1**, **2** and **3**). These data correspond to 16 DMF molecules (one DMF molecule per formula unit), which corresponds to 152–159 Å^3 and 33–36 electrons per DMF, in good agreement with one DMF molecule (supposed to occupy around 150 Å^3 and count on 40 electrons).

Therefore, the formulae for these MOFs may be estimated as $\{[M(\mu\text{-2ain})_2]\cdot DMF\}_n$.

S5. Thermogravimetric Analysis.

Compounds **1–3** show a very similar thermal response owing to their isostructural nature. The MOFs adsorb some amount of water (around one water molecule per formula unit) as confirmed by both thermogravimetric and elemental analysis results. In this regard, the fact that water is lost at so low temperature (below 60 ºC), is a clear evidence on the nature of water. This fact explains well the slight disagreement found when compared these results with the theoretical (crystallographic) formulae of the MOFs. TG curve of compound **1** (as a representative sample of the three MOFs) reveals that the loss of solvent (ca. one H_2O due to hydration of the MOFs and the lattice DMF molecule) takes place from room temperature up to 210 ºC, released in two separate stages. The high temperature required to liberate the lattice DMF molecule seems to indicate that it is somewhat occluded in the pores of the MOF. Almost immediately to the loss of solvent, the framework starts to decompose following two strong exothermic processes to lead to Co_3O_4 as final residue at *ca.*390 ºC. A more precise description of the thermogravimetric profile can be found in Table S2.

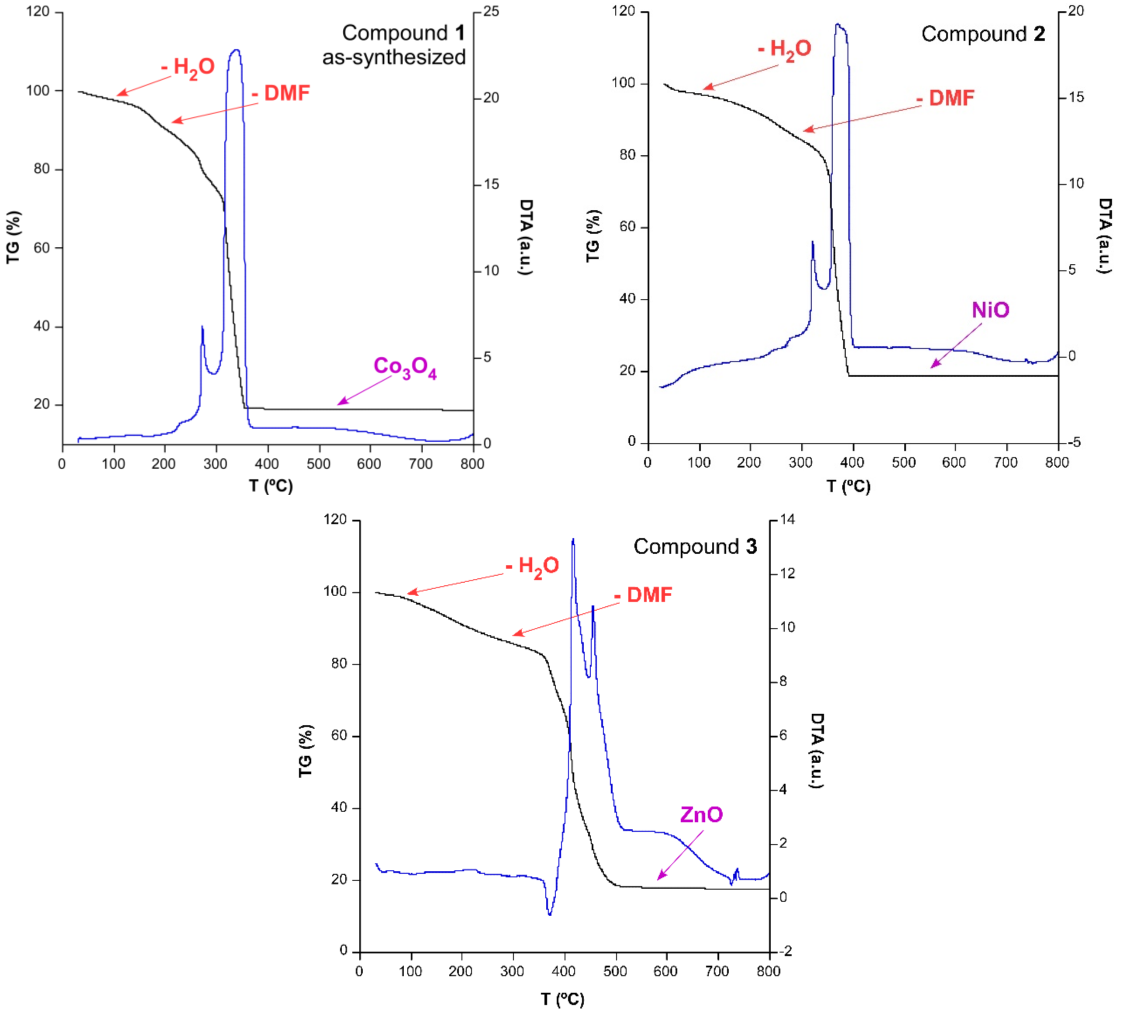

Figure S9. TG/DTA plots for compounds **1–3**.

Table S2. TG/DTA and elemental analysis data for compounds **1**–**3**.

$C_{15}H_{17}CoN_5O_5$ (compound 1) – hydrated		
**Ti–Tf**	**ΣΔm(%)**	**$ΣΔm(\%)_{teor}$**
30–65	3.0	3.0 (–0.7 x H_2O)
60–280	20.9	21.3 (–1 x DMF)
280–370	81.4	81.1 (–2 x 2ain)
370–800		Remaining residue (1/3 Co_3O_4)

Elemental analysis
Calcd.: C, 44.35; H, 4.22; Co, 14.51; N, 17.24. Found: C, 42.32; H, 4.38; Co, 13.83; N, 16.60.

$C_{15}H_{17}N_5NiO_5$ (compound 2) – hydrated		
**Ti–Tf**	**ΣΔm(%)**	**$ΣΔm(\%)_{teor}$**
30–65	3.0	3.0 (–0.7 x H_2O)
60–315	18.9	20.0 (–1 x DMF)
315–390	81.9	82.3 (–2 x 2ain)
390–800		Remaining residue (NiO)

Elemental analysis
Calcd.: C, 44.37; H, 4.22; N, 17.25; Ni, 14.45. Found: C, 42.65; H, 4.32; N, 16.45; Ni, 13.74.

$C_{15}H_{17}N_5O_5Zn$ (compound 3) – hydrated		
**Ti–Tf**	**ΣΔm(%)**	**$ΣΔm(\%)_{teor}$**
30–70	2.3	2.3 (–0.6 x H_2O)
70–350	18.7	19.2 (–1 x DMF)
350–530	80.9	81.1 (–2 x 2ain)
530–800		Remaining residue (ZnO)

Elemental analysis
Calcd.: C, 43.65; H, 4.15; N, 16.97; Zn, 15.84. Found: C, 43.25; H, 4.34; N, 16.41; Zn, 15.02.

Solvent exchange processes undertaken on compound **1** led to compounds **1-MeOH** and **1-DMSO**, which were analyzed by means of the same TG/DTA measurements. The solvent exchange can be clearly inferred from these plots, in which the different boiling points of the solvents modify the profiles of the TG curve. The loss of 2 MeOH and 1.5 DMSO molecules is in agreement with the rest of analyses carried out.

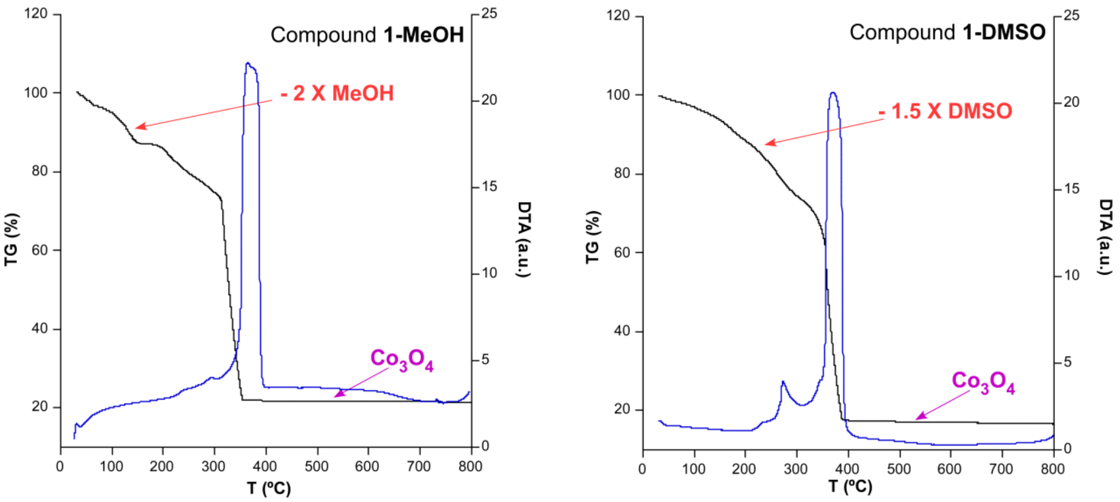

Figure S10. TG/DTA analysis forsolvent exchanged compounds **1-MeOH** and **1-DMSO**.

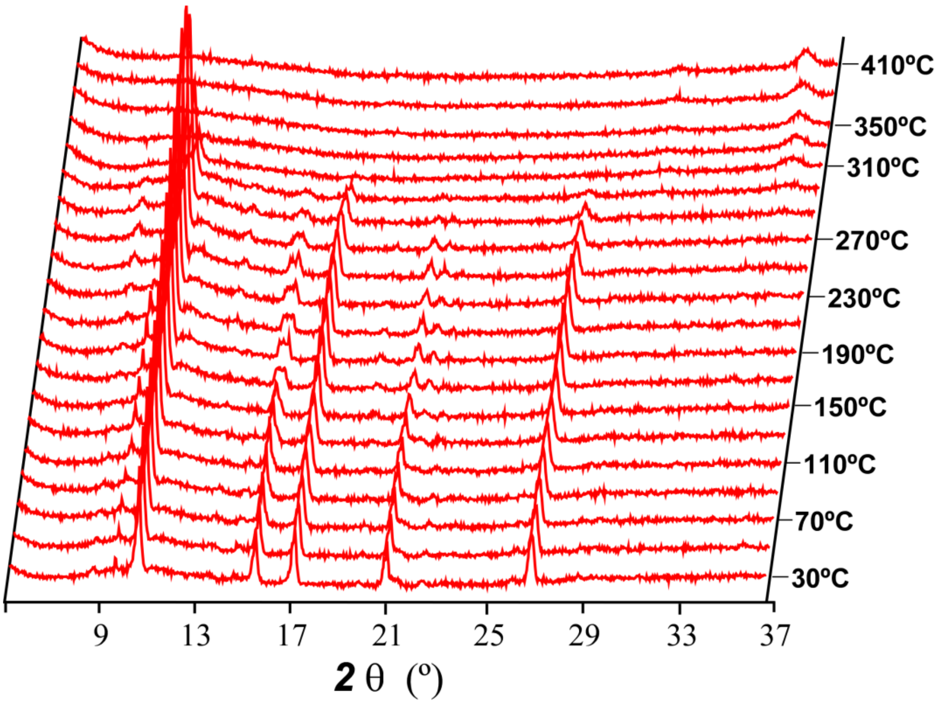

Figure S11. Thermodiffractometric analysis of **3**.

S6. FT-IR spectroscopy.

All compounds show a similar infrared spectrum which confirms the presence of the coordinated 2ain ligand. At high frequencies, the spectra exhibit an intense band at 3460 cm^{-1} that corresponds to the vibration of the O–H bond corresponding to thecrystallization water molecules, followed by weak vibrations at 3320 and 3220 cm^{-1} related with exocyclic amino group. Weak shoulders between 3100 and 2900 cm^{-1} are attributed to C–H vibrations of the pyridinic ring of the 2ain ligand. The intense vibrations in the 1660–1520 cm^{-1} region correspond to both the asymmetric stretching vibrations of the carboxylate groups and the aromatic C–C and C–N bonds, while the symmetric stretching vibrations of the carboxylate groups occur in the lower range of 1390–1270 cm^{-1}. At lower frequencies, the remaining bands are assigned to the distortions originated in the aromatic ring and the carboxylate groups of the 2ain ligand. The vibration bands of the M–O and M–N bonds are observed around 530 cm^{-1}.

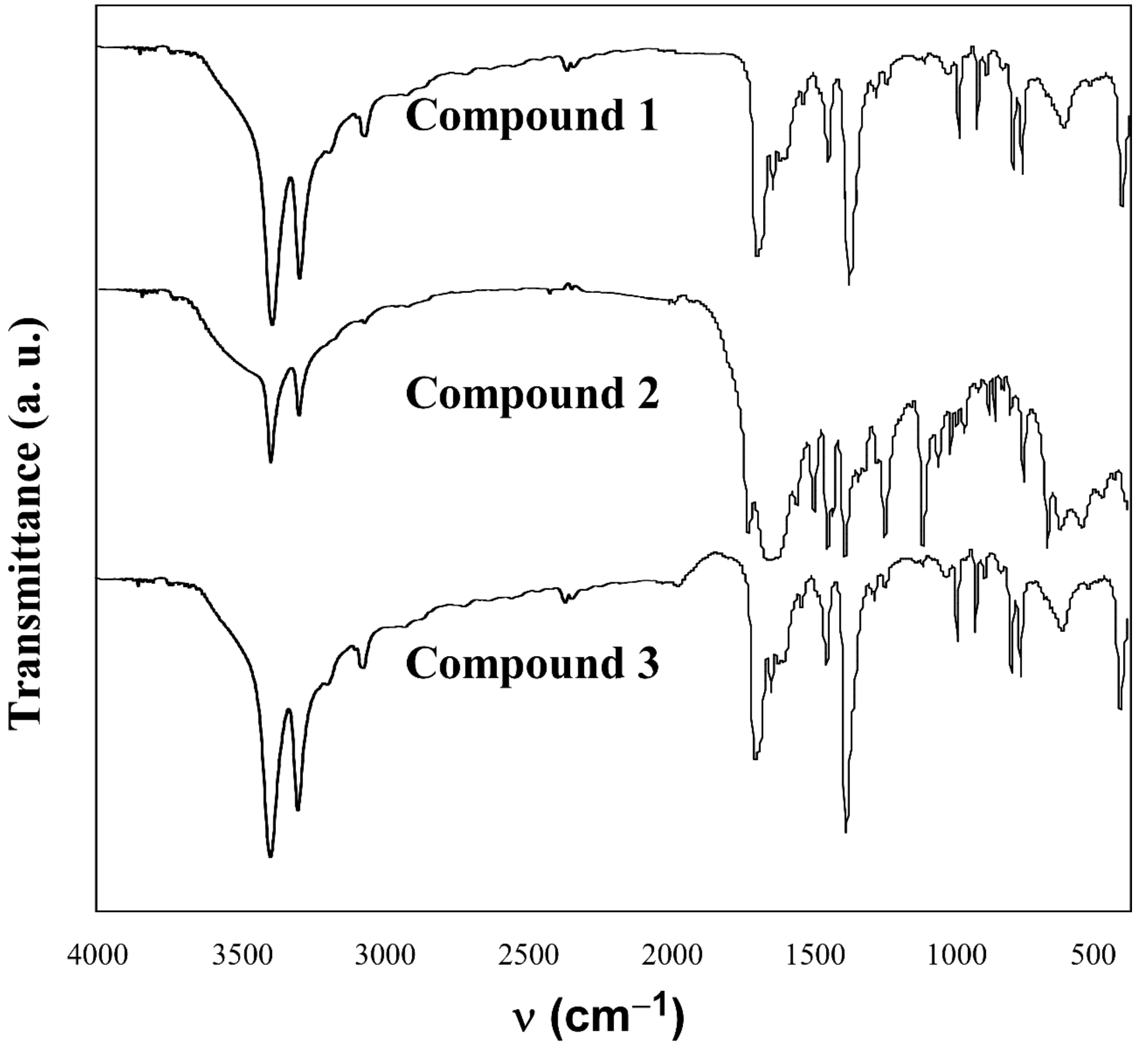

Figure S12. FTIR spectra of all compounds.

S7. *Dc* magnetic susceptibility measurements.

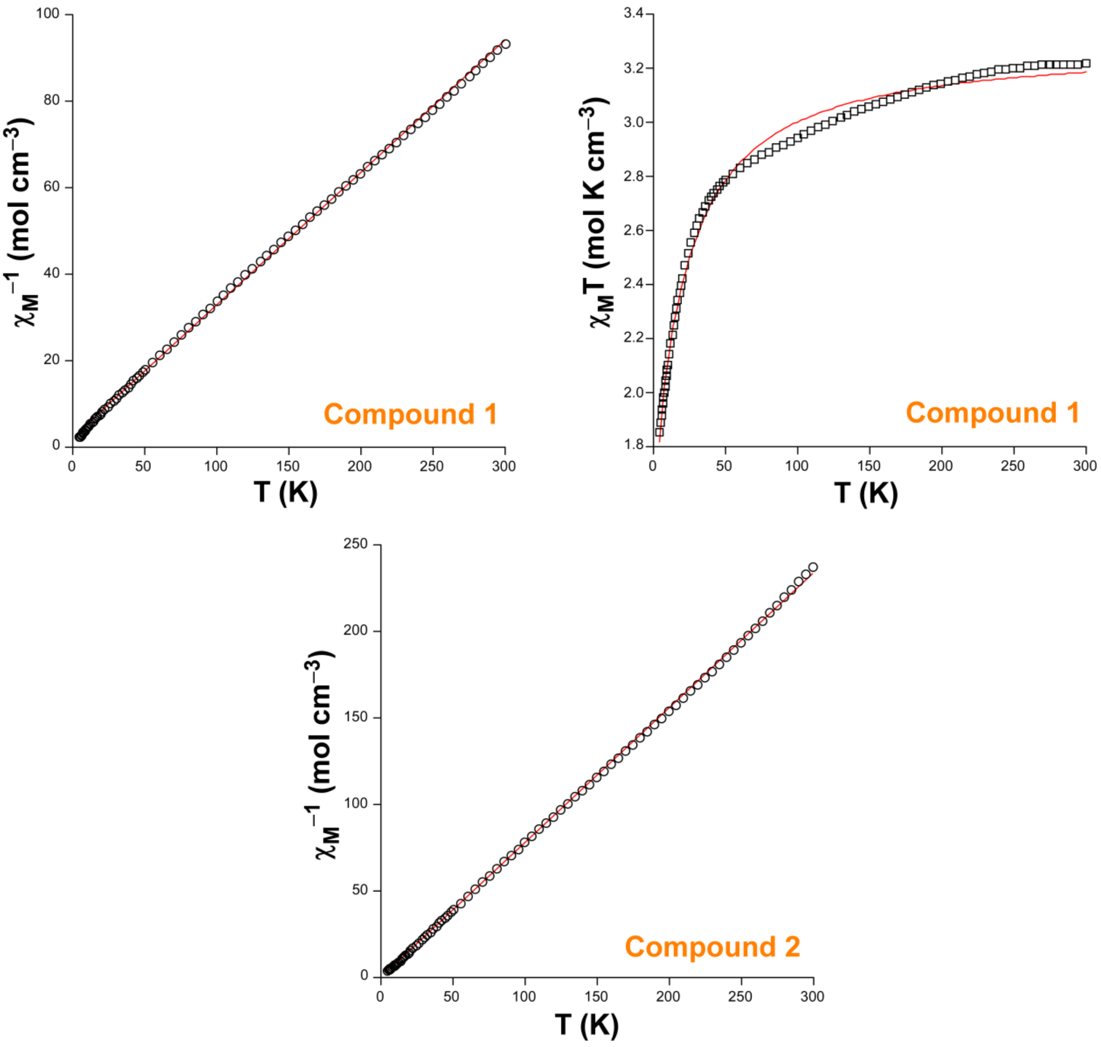

Figure S13. χ_M^{-1}vs T and $\chi_M T$ vs T plots of compounds **1** and **2** showing best theoretical fits (red line).

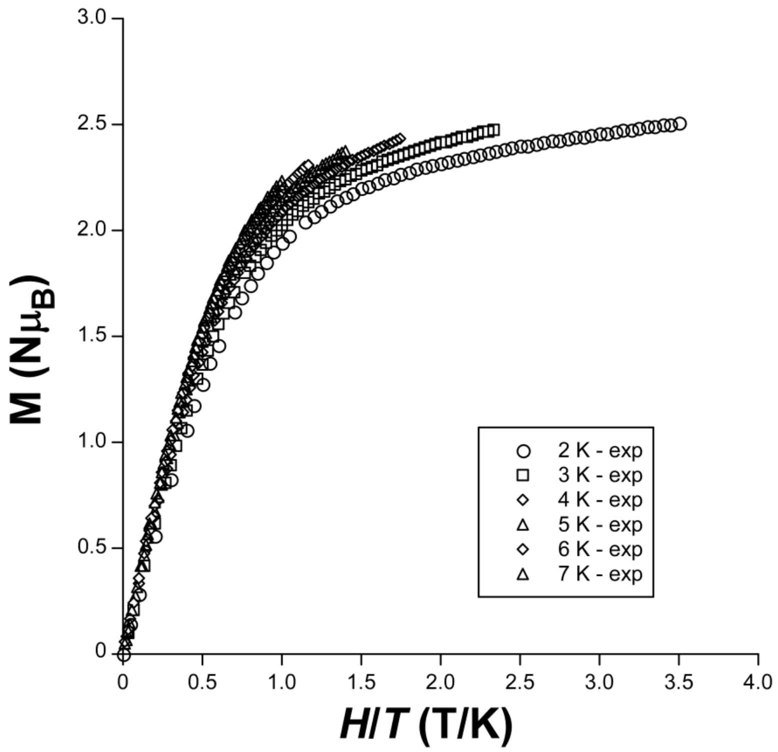

Figure S14. Variable temperature reduced magnetization curves for compound **1** under an applied field of 1000 Oe.

Dc properties of solvent-exchanged compounds 1-MeOH and 1-DMSO

When compound **1** is kept immersed in MeOH and DMSO for 2 days, it exchanges the solvent molecules originally trapped within the pores (one DMF and one H_2O molecules, in agreement with TG results of S4 section) by certain number of molecules of MeOH and DMSO. These two new compounds are found to possess the following formulae: $[Co(\mu\text{-}2ain)_2]\cdot 2MeOH$ (**1-MeOH**) and $[Co(\mu\text{-}2ain)_2]\cdot 1.5DMSO$ (**1-DMSO**).

A first inspection of the curves reveals that both compounds keep, to a large extent, a similar magnetic character of the pristine material, characterized for a significant magnetic anisotropy and/or the presence of weak antiferromagnetic coupling interactions between spin carriers. Best fitting results of the combined analysis of temperature dependent magnetic susceptibility and isothermal magnetization curves gives the following results shown in Table S3.

Table S3. Best fitting results of for compound **1-MeOH** and **1-DMSO**.

**Compound 1-MeOH**							
Hamiltonian SOC (eq 2)[b]							
λ	–117	σ	–1.20	Δ	211	g	2.21
Hamiltonian zfs (eq 3)[c]							
g_x / g_y	1.69	g_z	2.81	D	-16.0		
**Compound 1-DMSO**							
Hamiltonian SOC (eq 2)[b]							
λ	–119	σ	–1.23	Δ	198	g	2.13
Hamiltonian zfs (eq 3)[c]							
g_x / g_y	1.54	g_z	2.80	D	-20.0		

[a] Units: C constant and θ are given in cm^3 K mol^{-1} and K, respectively. [b] λ and Δparameters are expressed in cm^{-1}. [c] D parameter is given in cm^{-1}.

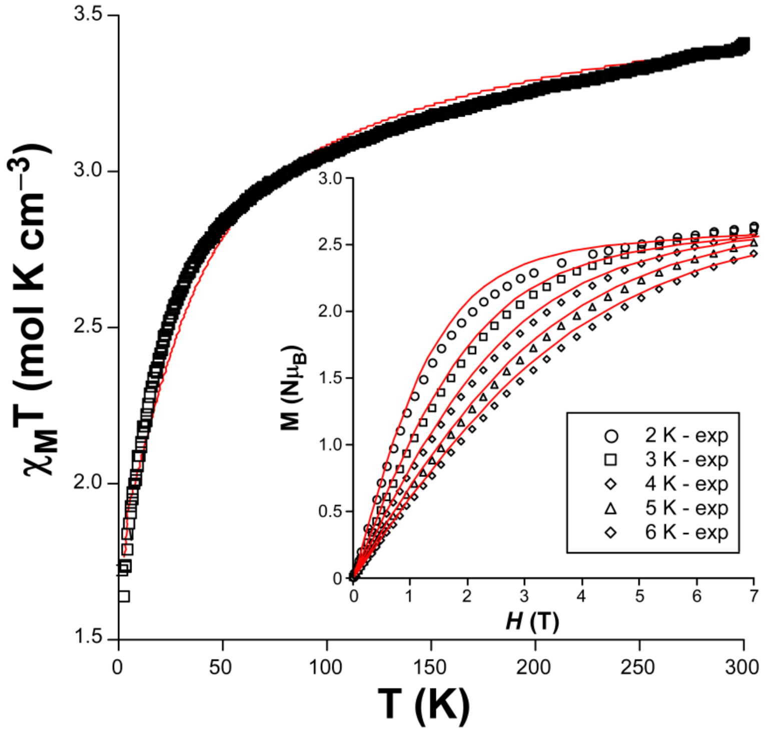

(a)

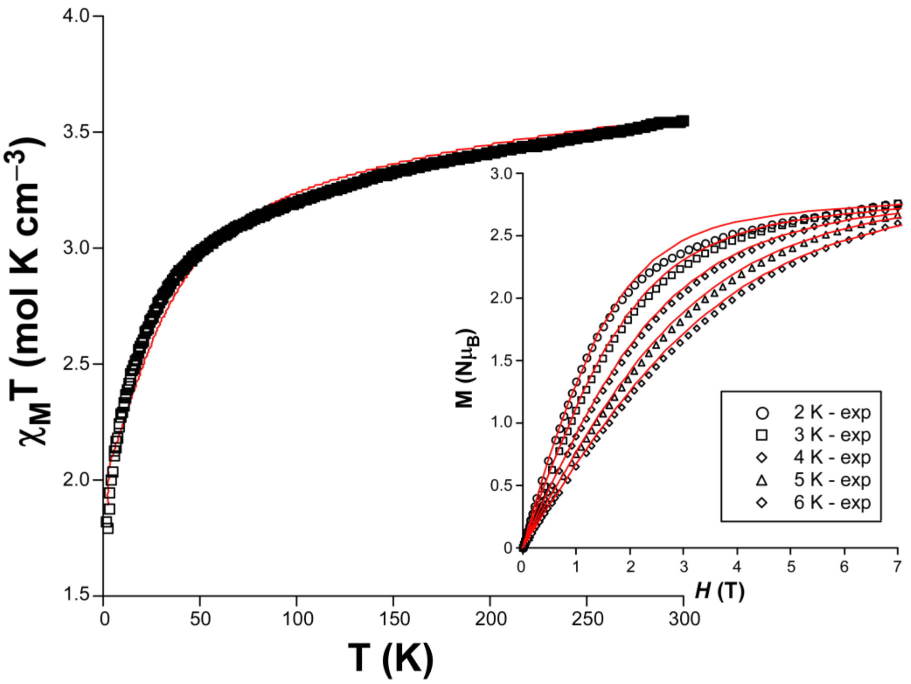

(b)

Figure S15. Variable temperature $\chi_M T$ vs $T$and M vs H plots with best fitting for **(a) 1-DMSO** and **(b) 1-MeOH** under an applied field of 1000 Oe.

S8. *Ac* magnetic susceptibility measurements.

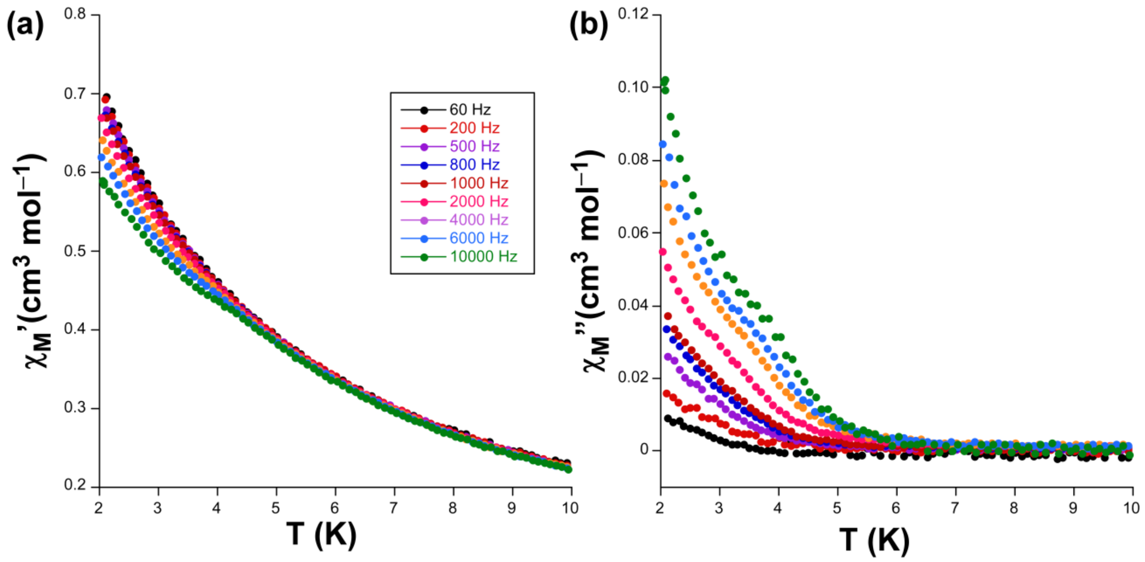

Figure S16. Temperature dependence of the **(a)** χ_M’ and **(b)** χ_M’’ signals for compound **1** with no applied *dc* field.

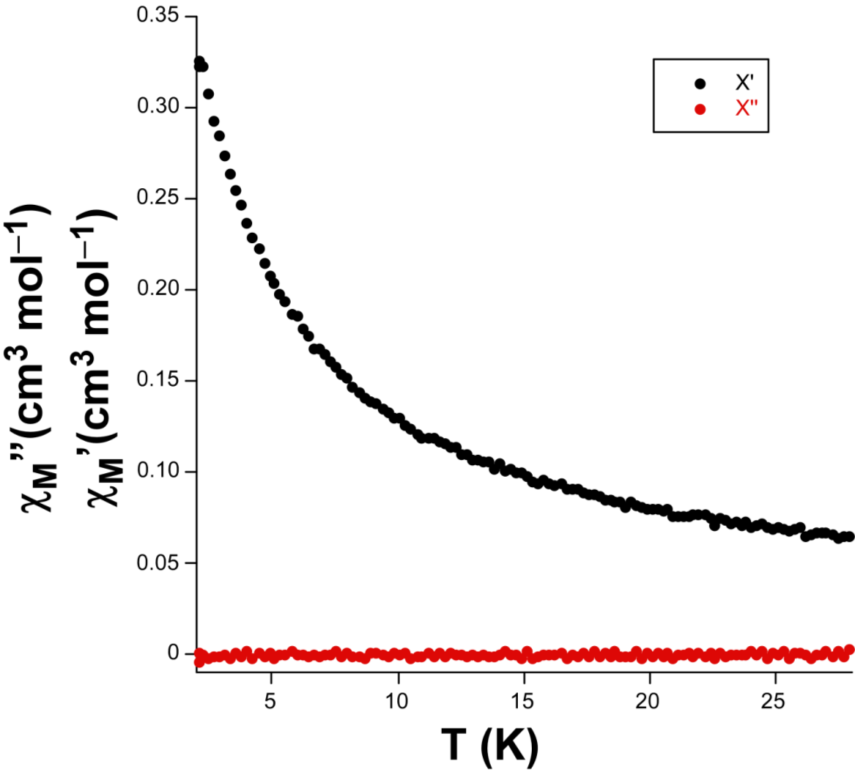

Figure S17. Temperature dependence of the χ_M’ and χ_M’’ signals for compound **2** under an applied field of 1000 Oe.

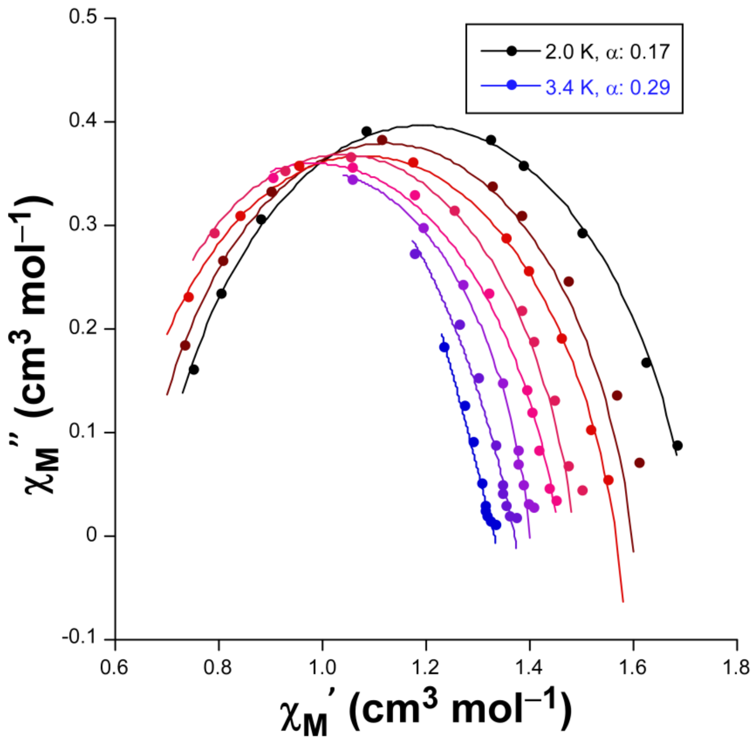

Figure S18. Cole-Cole plot for compound **1-DMSO** showing best fitting with Debye model.

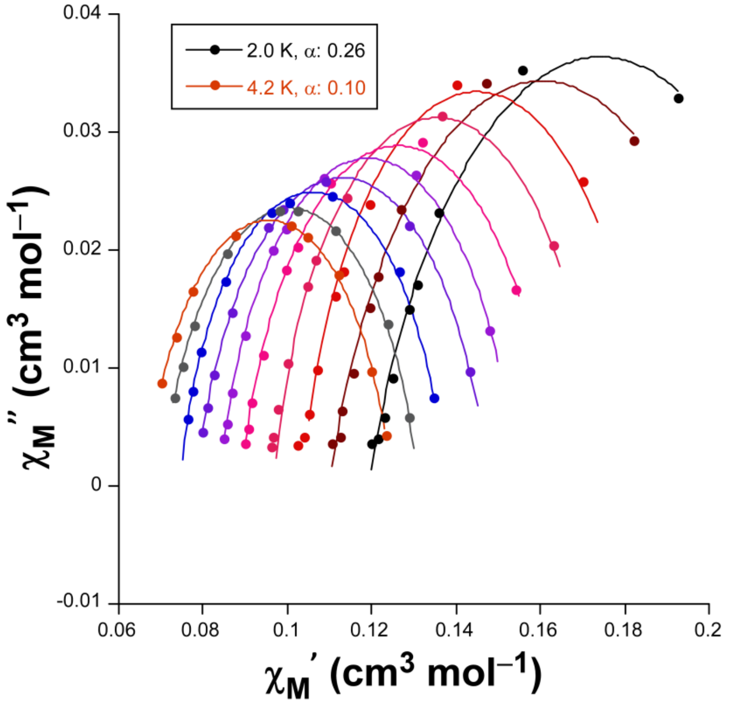

Figure S19. Cole-Cole plot for compound **1-MeOH** showing best fitting with Debye model.

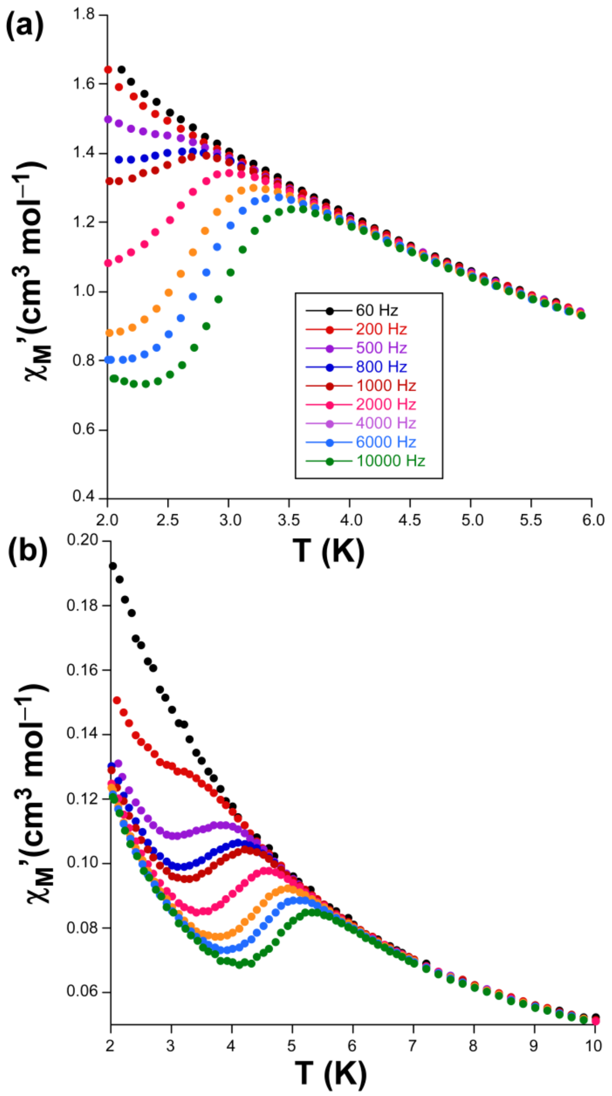

Figure S20. Temperature dependence of the χ′ signal for compounds **1-DMSO** and **1-MeOH**.

S9. Spin densities of broken symmetry calculations.

Calculations of the coupling constants through the broken symmetry strategy have been performed for compound **1**and **2**upon a suitable model based on a dimeric entitiy grown from crystallographic coordinates of X-ray crystal structure. Bridging and terminal ligands have been simplified replacing them by ammonia or formic acid.

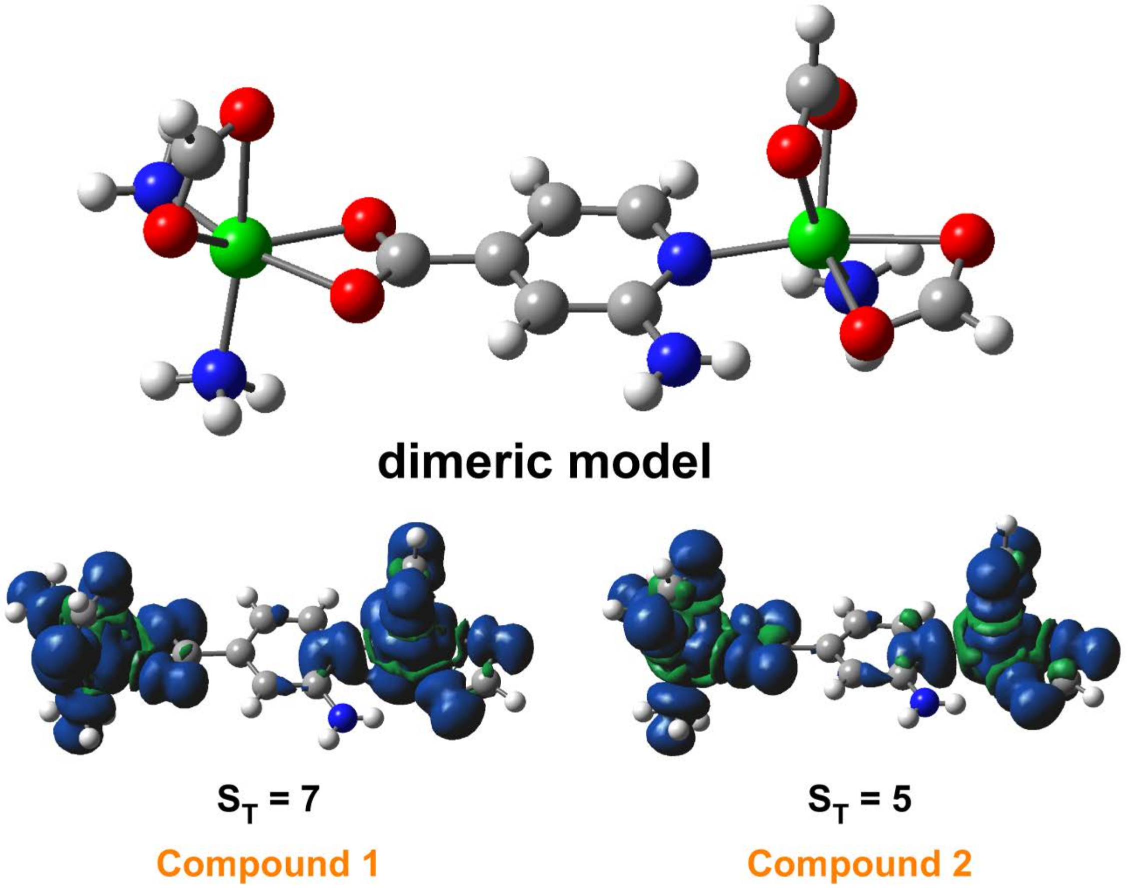

Figure S21. Model employed for calculation of *J* parameter and DFT high spin density distributions for models **1** and **2**.

S10. PL measurements of compound 3 on solid state.

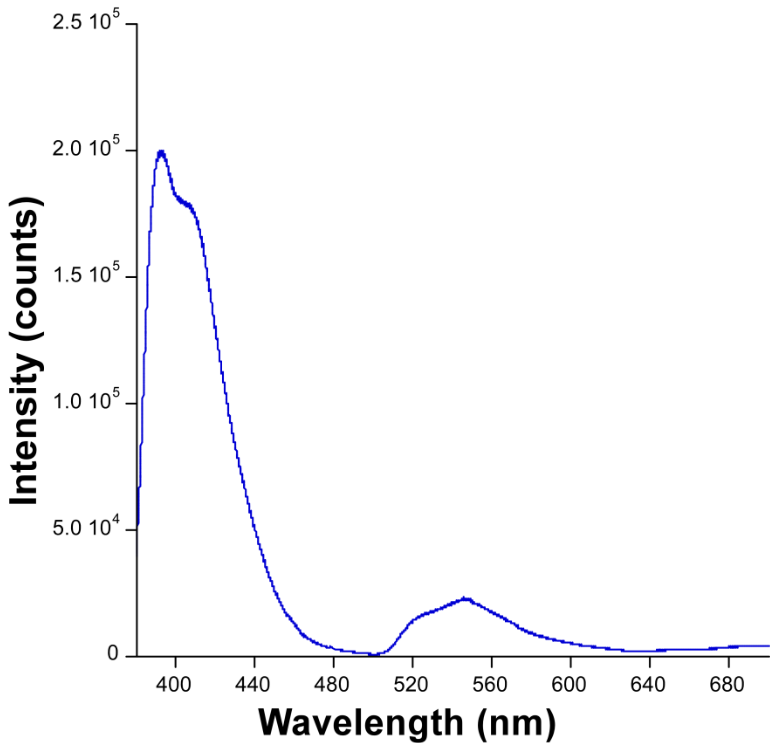

Figure S22. Emission spectrum of compound **3** under excitation at 370 nm.

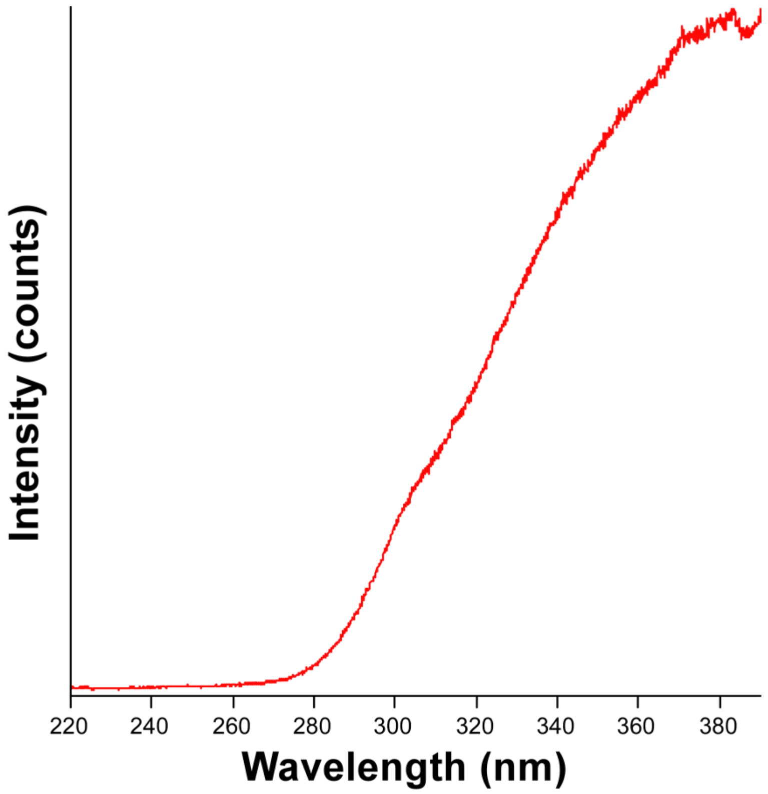

Figure S23. Excitation spectrum of compound **3** monitored under λ_{em} = 550 nm.

The PL quantum yield was measured at room temperature with an integrated sphere. In the following figure (Figure S25), both the full spectrum without and with sample may be observed.

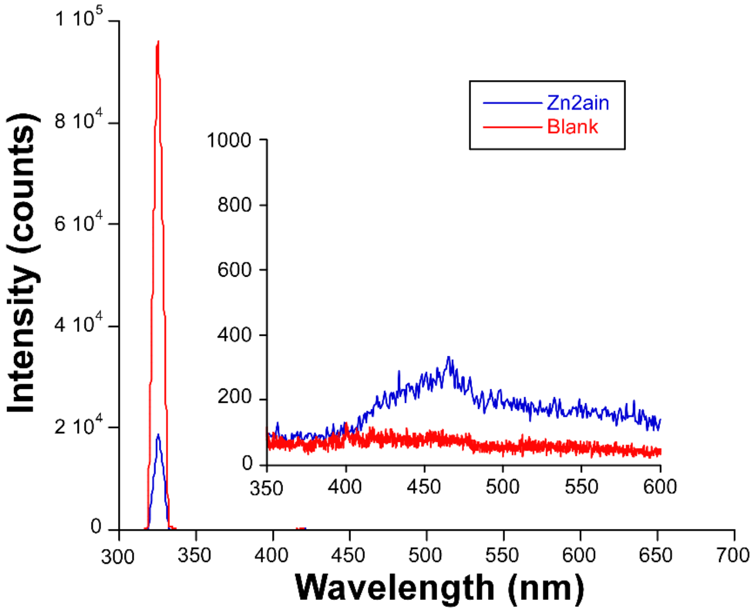

Figure S24. PL spectra employed for the calculation of the quantum yield.

With the aim of analyzing the evolution of the PL emission of compound **3** with temperature, the emission spectrum was measured cooling down the sample from room temperature down to 10 K. As observed in Figure S26, the emission intensity gets strongly enhanced below 200 ºC, describing the curve (based on integrated intensity shown on the right hand side). It must be remarked that the error in the integration of the data is quite low in such a way that it fits within the circles. The line connecting the circles does not correspond to any function but it only is a guide for the eye.

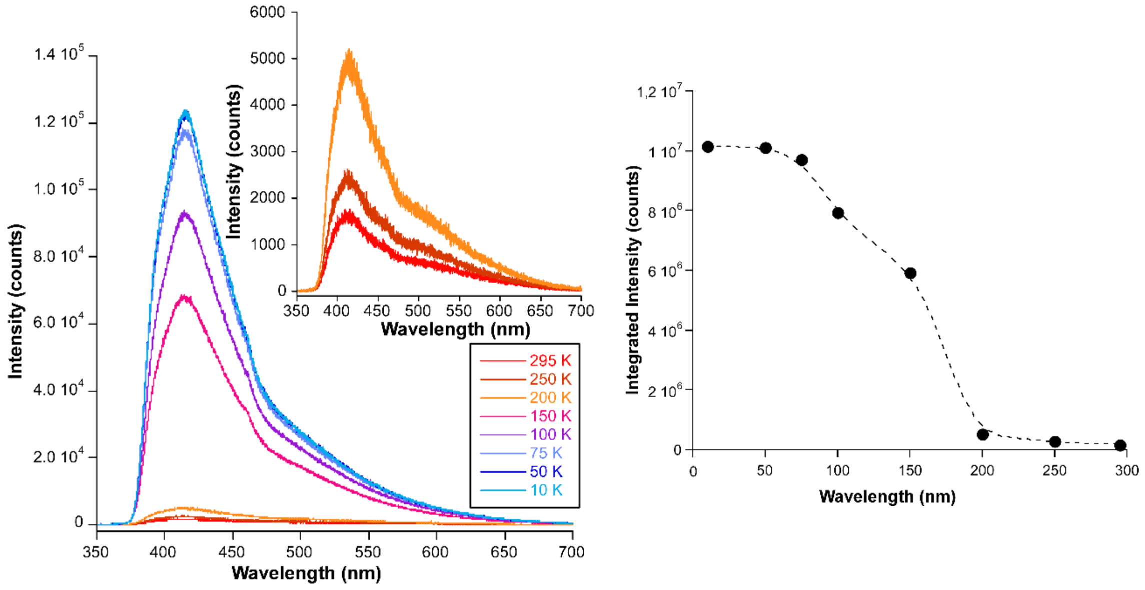

Figure S25. **(a)** Variable temperature emission spectra of **3** under excitation at 305 nm. **(b)** Evolution of the integrated intensity of the emission according to temperature.

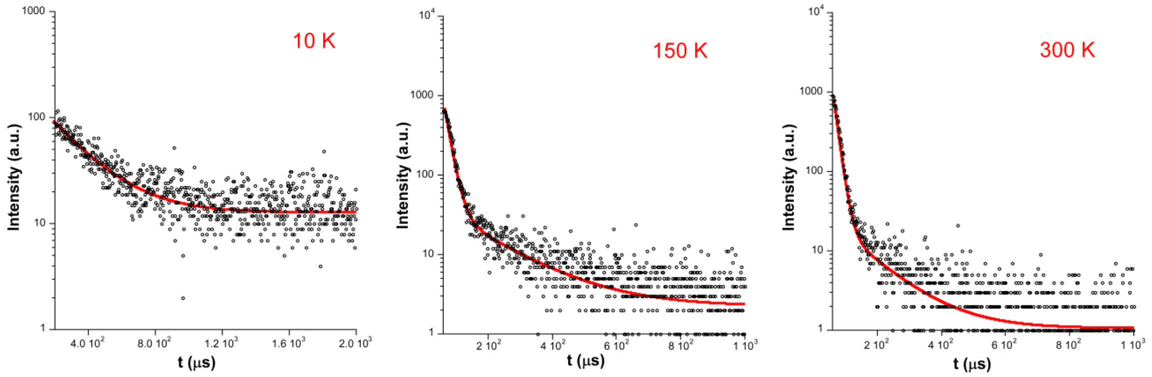

Figure S26. Decay curves with best fitting at selected temperatures for compound **3** (λ_{ex} = 305 and λ_{em} = 405 nm).

Lifetimes were fitted according to the exponential [$I_t = A_0 + A_1\exp(-t/\tau_1) + A_2\exp(-t/\tau_2)$] expression, where the shortest lifetimes recorded at 150 and 300 K correspond to the pulse of the microsecond lamp.

Table S4. Lifetime values and corresponding percentages for components obtained from best fittings of decay curves measured at room temperature for compound **1** (λ_{ex} = 325 nm).

Temperature (K)	λ(nm)	τ_1 (μs)	τ_2 (μs)	Chi Sq.
10	411	239(2)		1.118
150	411	18.1(1)	169(4)	1.182
300	411	14.1(1)	121(3)	1.175

S11. PL sensing properties of compound 3.

The excitation spectra were recorded for a dispersion consisting of 5 mg of compound **3** in 5 mL of the corresponding solvent, denoted solvent@**3** (see Figure S28). As observed, the solvent plays a crucial role since main excitation band suffers significant changes. Compared to solid state, H_2O and DMSO are the solvent causing the least significant change because the main excitation remain almost at the same wavelength [$\lambda_{max}(H_2O)$ = 357 nm and λ_{max}(DMSO) =365 nm vs λ_{max}(solid) = 370 nm], whereas the occurrence of new maximum (not present in solid state) peaking at 270 and 290 nm, respectively for H_2O and DMSO. The rest of solvents exhibit a unique excitation band that is largely shifted, showing values ranging between 320 (for MeOH) and 343 nm (for Ac_2O).

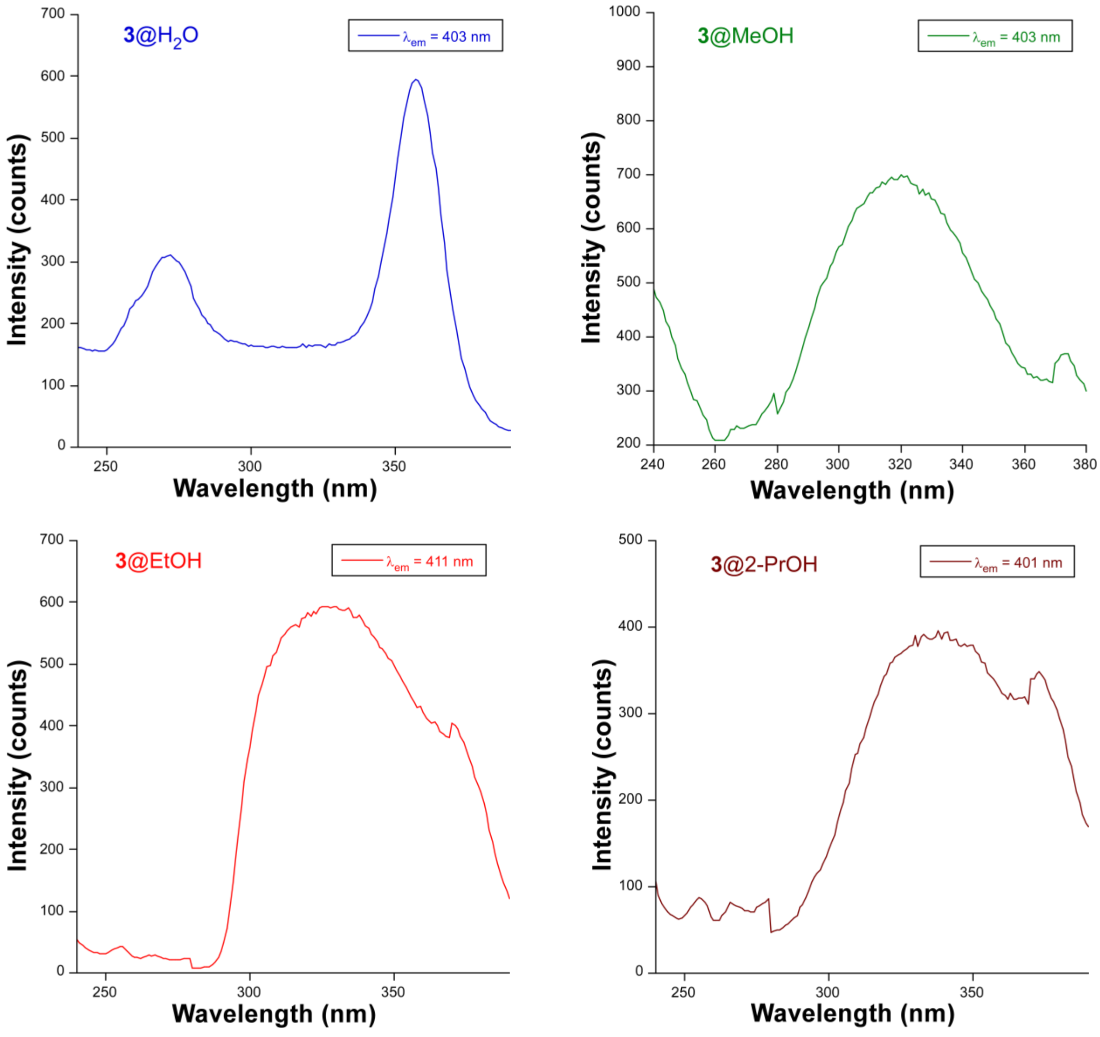

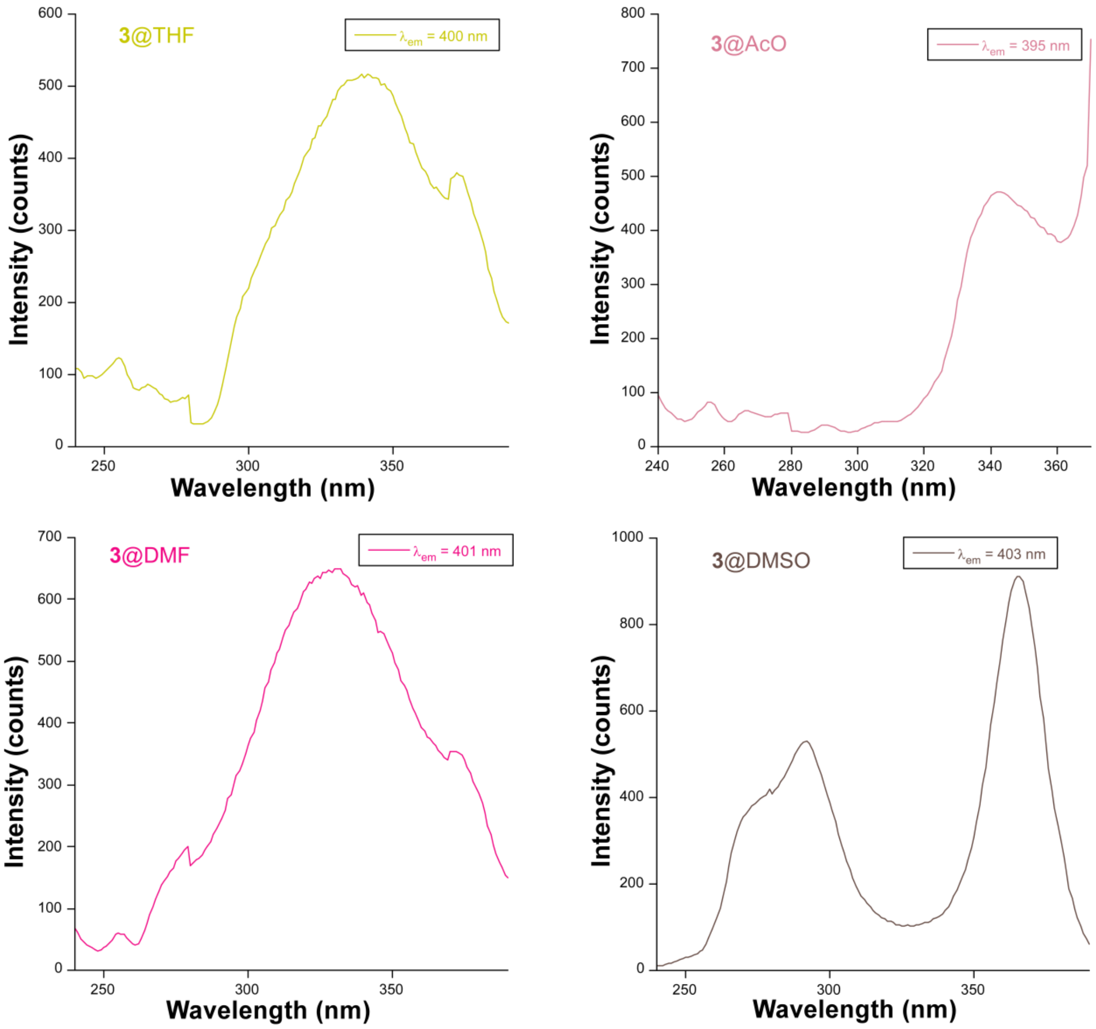

Figure S27. Excitation spectra of compound **3** dispersed on different solvents recorded with the same experimental conditions.

The shifts observed in the emission spectrum of each solvent are translated to the emission spectrum, where the λ_{em}ranges between 395 (for AcO) and 403 nm (for H_2O). More interestingly, the intensity of emission signal decreases following the sequence that follow the polarity.

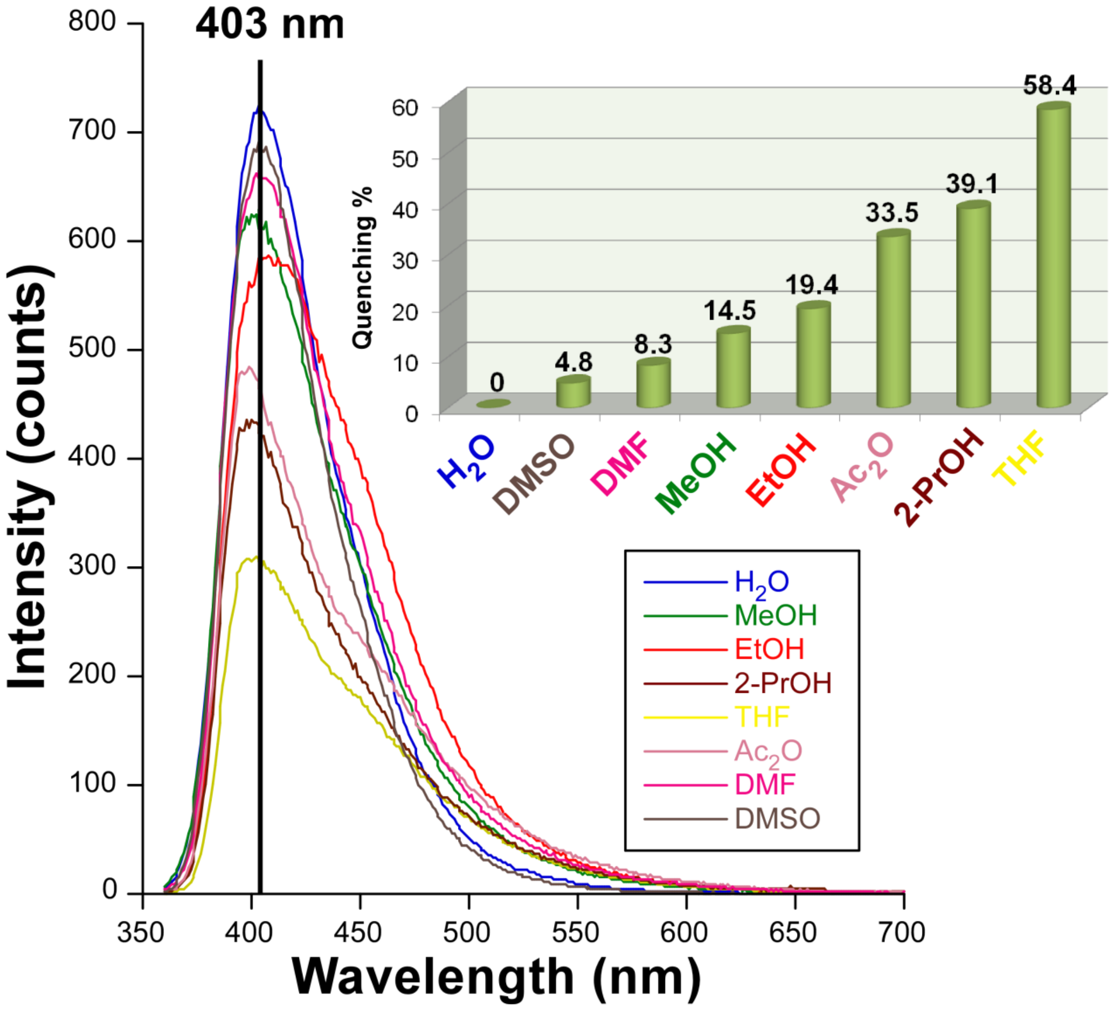

Figure S28. Quenching capacity of solvents based on the emission intensity of **3** under maximum excitation wavelength.

On another level, PL sensing properties of compound **3** in aqueous suspensions have been deeply analyzed for the detection of $Fe^{3+}$and Cu^{2+}given their large quenching compared to other studied ions. Stern-Volmer plot for Fe^{3+}concentration shows a non-linear curve indicating the simultaneous occurrence of dynamic and static quenching. Therefore, the curve has been fitted with 2nd order polynomial expression which accounts for both processes:[1]

$$I_0/I = (1 + k_{SV}[Q])(1 + k_a[Q]) = 1 + (k_{SV} + k_a)[Q]) + k_{SV}k_a[Q]^2 \qquad \text{(eq. 1)}$$

[1] (a) J. V. Goodpaster and V. L. McGuffin, *Appl. Spectrosc.*, 1999, **53**, 1000–1008. (b) J. Kusba, V. Bogdanov, I. Gryczynski and J. R. Lakowicz, *Biophys J.*, 1994, **5**, 2024–2040.

where k_{SV} and k_a stand for the constants involving dynamic and static quenching processes. Best fitting results for the polynomial expression give:

$$C = 0.47 \qquad (k_{SV} + k_a) = 3935 \qquad k_{SV}k_a = 7.94 \times 10^6$$

On the contrary, the linear dependence of the intensity according to the concentration of Cu^{2+} ions allows data be fitted to the Stern-Volmer equation:

$$I_0/I = 1 + k_{SV}[Q] \qquad \text{(eq. 2)}$$

The amino group of the 2ain ligands are exposed to the pores in such a way that they could interact with solvent or metal ions diffusing though the channel system of the MOF.

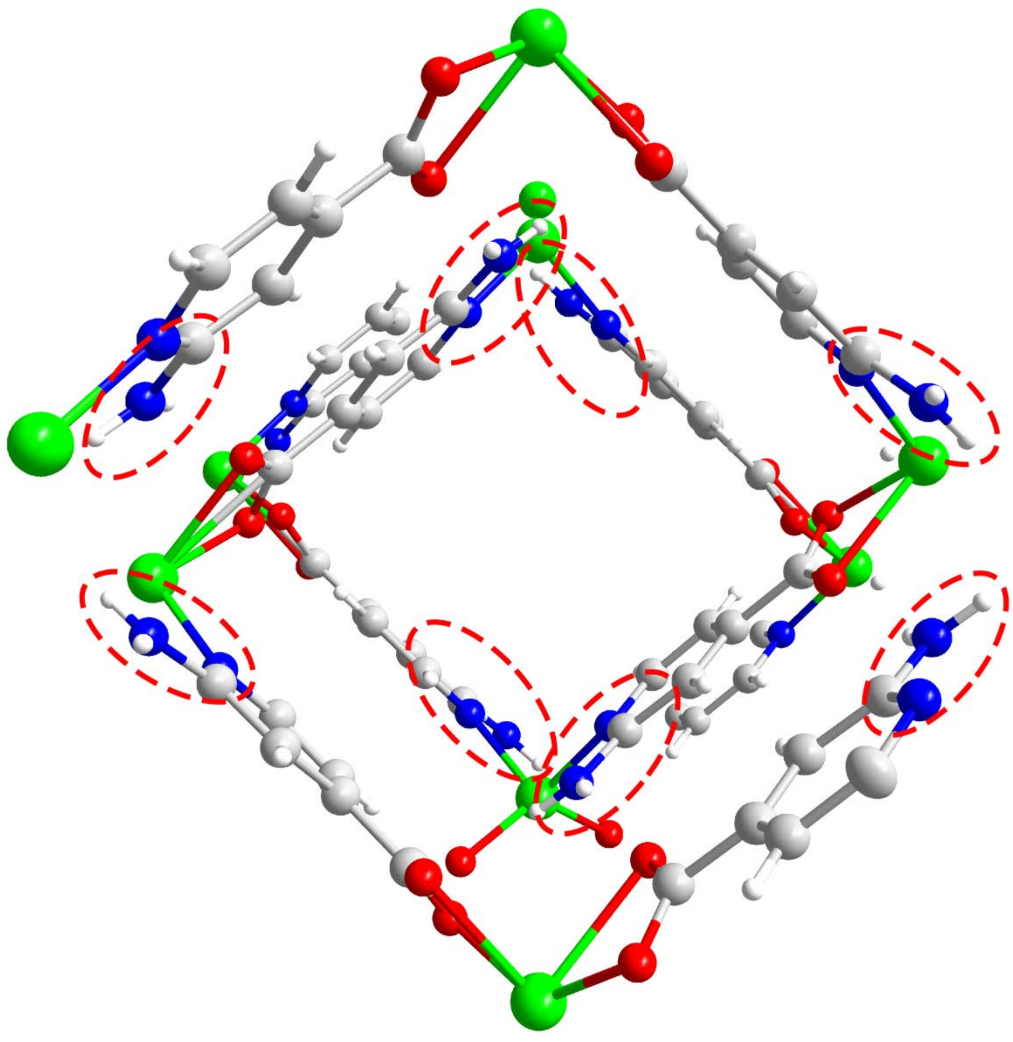

Figure S29. Excerpt of the structure of **3** showing the channel voids with the potential interaction sites (dashed circles).

Compound **3** was also analyzed for specific sensing between the strongest quenchers (Fe^{3+} and Cu^{2+}). As observed in the following figure, the quenching rate is not exactly proportional to that derived from the sum of both isolated quenchers, but it corresponds to a different scenario.

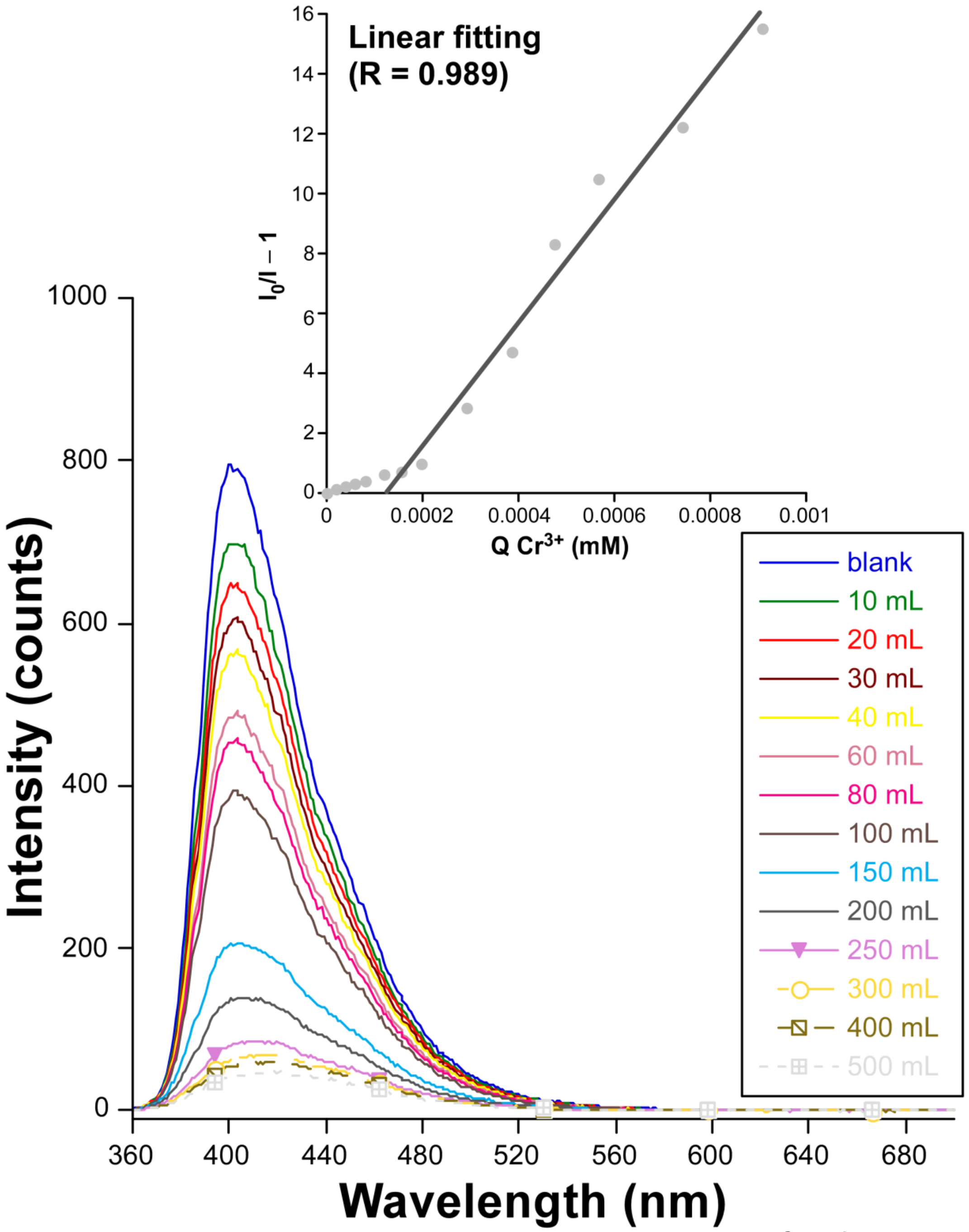

Figure S30. Emission spectra of **3** and Stern-Volmer plot for a mixed Fe^{3+}/Cu^{2+} solution.

Finally, the integrity of compound **3** was also analyzed after dispersing the solid sample in solvents. As shown in Figure S31, the samples filtered from the corresponding suspension exhibit an almost undistinguishable pattern, which corroborates structural integrity is maintained during the experiment. The same experiment was also performed for samples suspended in aqueous solutions of Fe^{3+} and Cu^{2+}. PXRD measurements

performed on recovered solids show a similar pattern as well, a fact that also excludes any possible replacement.

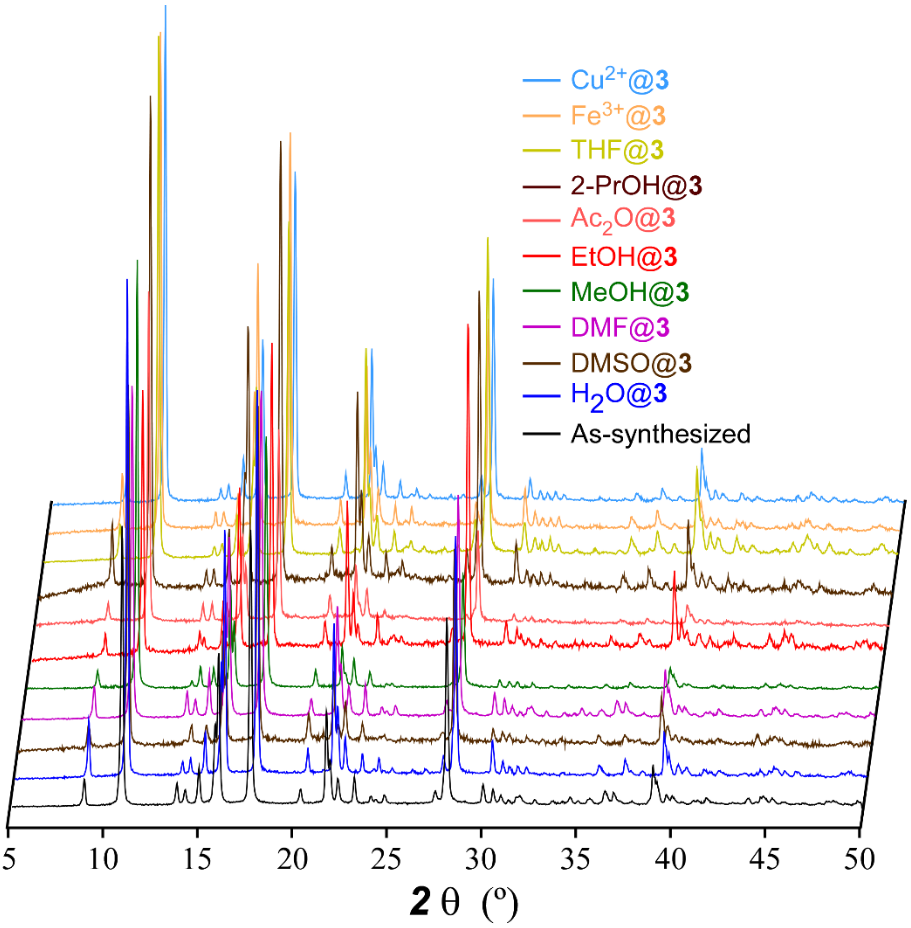

Figure S31. PXRD data of as-synthesized sample and samples collected after suspensions.
