# Supplementary material for: Magnetic and Photoluminescent Sensors Based on Metal-Organic Frameworks Built up from 2-aminoisonicotinate
Source: Sci Rep. 2020 Jun 1;10:8843. doi: 10.1038/s41598-020-65687-6 (PMC7264304; doi:10.1038/s41598-020-65687-6)

checkCIF/PLATON report

Structure factors have been supplied for datablock(s) Compound3

THIS REPORT IS FOR GUIDANCE ONLY. IF USED AS PART OF A REVIEW PROCEDURE FOR PUBLICATION, IT SHOULD NOT REPLACE THE EXPERTISE OF AN EXPERIENCED CRYSTALLOGRAPHIC REFEREE.

No syntax errors found. CIF dictionary Interpreting this report

Datablock: Compound3

Bond precision: C-C = 0.0030 A Wavelength=0.71073

Cell:	a=12.9026(9)	b=22.8687(15)	c=23.8896(16)
	alpha=90	beta=90	gamma=90
Temperature:	100 K		

	Calculated	Reported
Volume	7049.0(8)	7049.0(8)
Space group	F d d d	F d d d
Hall group	-F 2uv 2vw	-F 2uv 2vw
Moiety formula	C12 H10 N4 O4 Zn [+ solvent]	C12 H10 N4 O4 Zn, (C3 H7 N O)
Sum formula	C12 H10 N4 O4 Zn [+ solvent]	C15 H17 N5 O5 Zn
Mr	339.63	412.72
Dx,g cm-3	1.280	1.623
Z	16	16
Mu (mm-1)	1.410	1.438
F000	2752.0	3552.0
F000’	2757.57	
h,k,lmax	17,30,32	17,30,31
Nref	2247	2121
Tmin,Tmax	0.902,0.931	0.914,0.991
Tmin’	0.891	

Correction method= # Reported T Limits: Tmin=0.914 Tmax=0.991
AbsCorr = NUMERICAL

Data completeness= 0.944 Theta(max)= 28.497

R(reflections)= 0.0303(1764) wR2(reflections)= 0.0835(2121)

S = 1.077 Npar= 96

The following ALERTS were generated. Each ALERT has the format
test-name_ALERT_alert-type_alert-level.
Click on the hyperlinks for more details of the test.

Alert level C

```
DENSD01_ALERT_1_C  The ratio of the submitted crystal density and that
            calculated from the formula is outside the range 0.99 <> 1.01
            Crystal density given    =      1.623
            Calculated crystal density =      1.555
PLAT046_ALERT_1_C Reported Z, MW and D(calc) are Inconsistent ....      1.556 Check
```

Alert level G

```
FORMU01_ALERT_2_G  There is a discrepancy between the atom counts in the
            _chemical_formula_sum and the formula from the _atom_site* data.
            Atom count from _chemical_formula_sum:C15 H17 N5 O5 Zn1
            Atom count from the _atom_site data:  C12 H10 N4 O4 Zn1
CELLZ01_ALERT_1_G Difference between formula and atom_site contents detected.
CELLZ01_ALERT_1_G ALERT: Large difference may be due to a
            symmetry error - see SYMMG tests
           From the CIF: _cell_formula_units_Z   16
           From the CIF: _chemical_formula_sum  C15 H17 N5 O5 Zn
           TEST: Compare cell contents of formula and atom_site data

           atom    Z*formula  cif sites diff
           C        240.00    192.00   48.00
           H        272.00    160.00  112.00
           N         80.00     64.00   16.00
           O         80.00     64.00   16.00
           Zn        16.00     16.00    0.00
PLAT004_ALERT_5_G Polymeric Structure Found with Maximum Dimension          3 Info
PLAT007_ALERT_5_G Number of Unrefined Donor-H Atoms ..............          2 Report
PLAT014_ALERT_1_G N.O.K.   _shelx_fab_checksum Found in CIF ......     Please Check
PLAT041_ALERT_1_G Calc. and Reported SumFormula    Strings  Differ     Please Check
PLAT068_ALERT_1_G Reported F000 Differs from Calcd (or Missing)...     Please Check
PLAT605_ALERT_4_G Largest Solvent Accessible VOID in the Structure        319 A**3
PLAT720_ALERT_4_G Number of Unusual/Non-Standard Labels ..........          2 Note
PLAT764_ALERT_4_G Overcomplete CIF Bond List Detected (Rep/Expd) .       1.13 Ratio
PLAT794_ALERT_5_G Tentative Bond Valency for Zn1       (II)      .       1.86 Info
PLAT869_ALERT_4_G ALERTS Related to the Use of SQUEEZE Suppressed           ! Info
PLAT912_ALERT_4_G Missing # of FCF Reflections Above STh/L=  0.600        124 Note
PLAT978_ALERT_2_G Number C-C Bonds with Positive Residual Density.          4 Info
```

---

0 **ALERT level A** = Most likely a serious problem - resolve or explain
0 **ALERT level B** = A potentially serious problem, consider carefully
2 **ALERT level C** = Check. Ensure it is not caused by an omission or oversight
15 **ALERT level G** = General information/check it is not something unexpected

7 ALERT type 1 CIF construction/syntax error, inconsistent or missing data
2 ALERT type 2 Indicator that the structure model may be wrong or deficient
0 ALERT type 3 Indicator that the structure quality may be low
5 ALERT type 4 Improvement, methodology, query or suggestion
3 ALERT type 5 Informative message, check

It is advisable to attempt to resolve as many as possible of the alerts in all categories. Often the minor alerts point to easily fixed oversights, errors and omissions in your CIF or refinement strategy, so attention to these fine details can be worthwhile. In order to resolve some of the more serious problems it may be necessary to carry out additional measurements or structure refinements. However, the purpose of your study may justify the reported deviations and the more serious of these should normally be commented upon in the discussion or experimental section of a paper or in the "special_details" fields of the CIF. checkCIF was carefully designed to identify outliers and unusual parameters, but every test has its limitations and alerts that are not important in a particular case may appear. Conversely, the absence of alerts does not guarantee there are no aspects of the results needing attention. It is up to the individual to critically assess their own results and, if necessary, seek expert advice.

Publication of your CIF in IUCr journals

A basic structural check has been run on your CIF. These basic checks will be run on all CIFs submitted for publication in IUCr journals (*Acta Crystallographica*, *Journal of Applied Crystallography*, *Journal of Synchrotron Radiation*); however, if you intend to submit to *Acta Crystallographica Section C* or *E* or *IUCrData*, you should make sure that full publication checks are run on the final version of your CIF prior to submission.

Publication of your CIF in other journals

Please refer to the *Notes for Authors* of the relevant journal for any special instructions relating to CIF submission.

PLATON version of 22/12/2019; check.def file version of 13/12/2019

Datablock Compound3 - ellipsoid plot

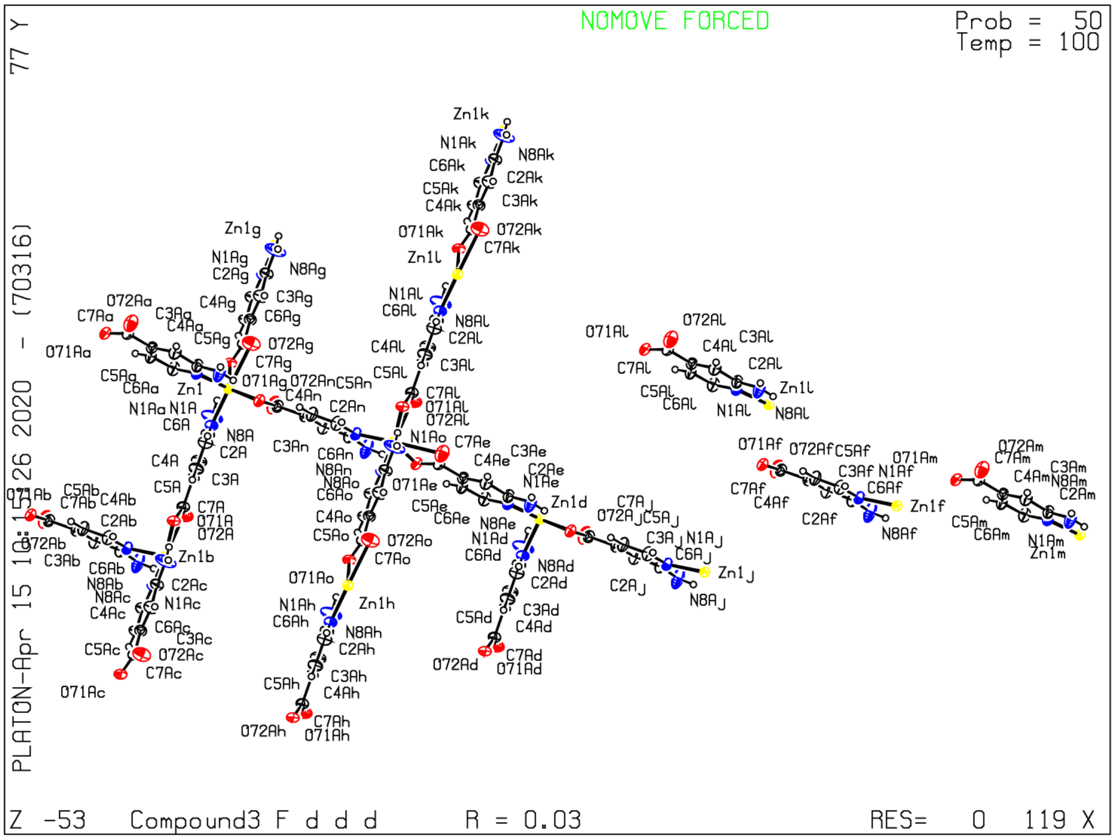

Supplement: Supplementary file 4 — checkcif_Compound1.pdf [file 41598_2020_65687_MOESM4_ESM.pdf]
